# Structural Manipulations of Marine Natural Products Inspire a New Library of 3-Amino-1,2,4-Triazine PDK Inhibitors Endowed with Antitumor Activity in Pancreatic Ductal Adenocarcinoma

**DOI:** 10.3390/md21050288

**Published:** 2023-05-04

**Authors:** Daniela Carbone, Michele De Franco, Camilla Pecoraro, Davide Bassani, Matteo Pavan, Stella Cascioferro, Barbara Parrino, Girolamo Cirrincione, Stefano Dall’Acqua, Stefania Sut, Stefano Moro, Valentina Gandin, Patrizia Diana

**Affiliations:** 1 Department of Biological, Chemical, and Pharmaceutical Sciences and Technologies (STEBICEF), University of Palermo, Via Archirafi 32, 90123 Palermo, Italy; daniela.carbone@unipa.it (D.C.); camilla.pecoraro@unipa.it (C.P.); stellamaria.cascioferro@unipa.it (S.C.); barbara.parrino@unipa.it (B.P.); girolamo.cirrincione@unipa.it (G.C.); 2 Department of Pharmaceutical and Pharmacological Sciences, University of Padova, Via Marzolo 5, 35128 Padova, Italy; michele.defranco@studenti.unipd.it (M.D.F.); stefano.dallacqua@unipd.it (S.D.); stefania.sut@studenti.unipd.it (S.S.); 3 Molecular Modeling Section (MMS), Department of Pharmaceutical and Pharmacological Sciences, University of Padova, 35131 Padova, Italy; davide.bassani.1@phd.unipd.it (D.B.); matteo.pavan.7@phd.unipd.it (M.P.); stefano.moro@unipd.it (S.M.)

**Keywords:** pancreatic ductal adenocarcinoma (PDAC), nortopsentin analogues, antitumor activity, pyruvate dehydrogenase kinases (PDKs), cytotoxic activity, metabolic alterations, ligand-based homology modeling, KRAS

## Abstract

Pancreatic ductal adenocarcinoma (PDAC) is one of the main aggressive types of cancer, characterized by late prognosis and drug resistance. Among the main factors sustaining PDAC progression, the alteration of cell metabolism has emerged to have a key role in PDAC cell proliferation, invasion, and resistance to standard chemotherapeutic agents. Taking into account all these factors and the urgency in evaluating novel options to treat PDAC, in the present work we reported the synthesis of a new series of indolyl-7-azaindolyl triazine compounds inspired by marine bis-indolyl alkaloids. We first assessed the ability of the new triazine compounds to inhibit the enzymatic activity of pyruvate dehydrogenase kinases (PDKs). The results showed that most of derivatives totally inhibit PDK1 and PDK4. Molecular docking analysis was executed to predict the possible binding mode of these derivatives using ligand-based homology modeling technique. Evaluation of the capability of new triazines to inhibit the cell growth in 2D and 3D KRAS-wild-type (BxPC-3) and KRAS-mutant (PSN-1) PDAC cell line, was carried out. The results showed the capacity of the new derivatives to reduce cell growth with a major selectivity against KRAS-mutant PDAC PSN-1 on both cell models. These data demonstrated that the new triazine derivatives target PDK1 enzymatic activity and exhibit cytotoxic effects on 2D and 3D PDAC cell models, thus encouraging further structure manipulation for analogs development against PDAC.

## 1. Introduction

Cancer cells rewire many metabolic pathways to sustain their survival, growth, invasiveness and resistance to cancer treatments [1].

Pyruvate dehydrogenase complex (PDC) has emerged as a key enzyme in the regulation of metabolic pathway of glucose. In normal human cells, PDC catalyzes the decarboxylation of the pyruvate in acetyl CoA and carbon dioxide—a process often deregulated in cancer cells, in which aerobic glycolysis is used to produce ATP instead of oxidative phosphorylation (OXPHOS), even in presence of adequate oxygen supply [2]. This phenomenon is well known as the “Warburg effect” and provides some tangible advantages to cancer survival, such as a high rate of ATP production, low immunity microenvironment and enhanced cancer cell invasion [3].

The pyruvate dehydrogenase kinases (PDKs) have been identified as the leading players of this metabolic swing since they are involved in PDC inactivation [4,5].

Four PDK isoforms were isolated in humans (PDK1, PDK2, PDK3, PDK4) [6] and their overexpression was frequently associated with the KRAS-mutated cancer types, such as pancreatic ductal adenocarcinoma (PDAC) and colorectal cancer [7,8].

PDAC adopts a distinct metabolic process to meet the energetic requests of growing cancer cells [9] which is clearly associated with chemo-, radio- and immunotherapy resistance [10,11,12].

Therefore, there is an urgent need to develop novel therapeutic strategies to treat PDAC, and the enzymes involved in metabolic alterations, such as PDKs, can be considered valuable targets to develop efficacious anticancer strategies [13,14].

Considering this aim and that approximately 80% of the approved chemotherapeutic drugs are based on bioactive natural products [15,16], the marine microenvironment has been exploited as a huge resource of novel-drug-lead compounds. 

**Figure 1:**
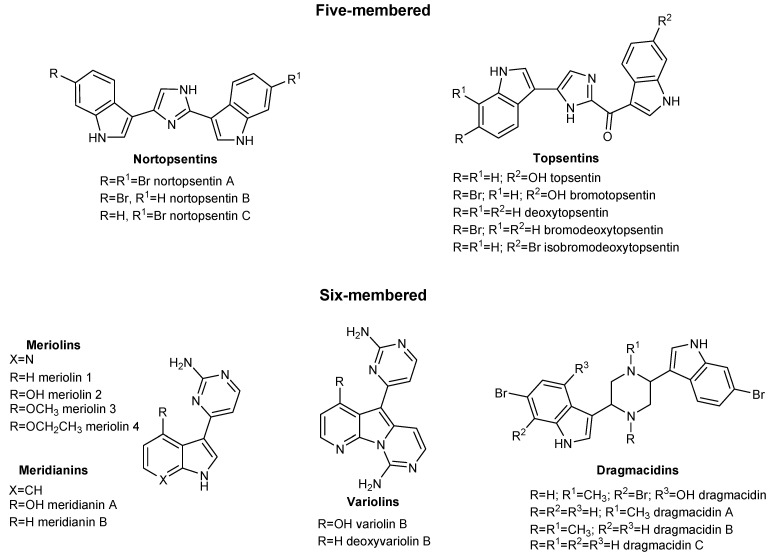
Five- and six-membered natural compounds isolated from marine sponges.

Many marine alkaloids, including nortopsentin, topsentin [17,18], meridianins, variolins, meriolins and dragmacidins [19,20], reported in Figure 1, have been described for their antiproliferative activity often due to their ability in inhibiting different kinases.

We have previously synthetized a library of marine analogs, replacing the central imidazole ring of nortopsentin and topsentin with several five-membered heterocycles or by manipulation of their indolyl portion [21,22,23,24,25,26].

Many of these derivatives have been proven to be extremely potent against a wide range of pancreatic cancer cells, hampering the activity of different protein kinases heavily involved in cancer progression [27,28].

Specifically, the series of the indolyl thiazolyl 7-azaindole nortopsentin analogs showed a good level of antiproliferative activity in the micro-submicromolar range and cyclin-dependent kinase 1 (CDK1) inhibition with IC_50_ values of 0.64–0.89 µM [28]. Similarly, structural manipulations of topsentin, replacing the imidazole central core with 1,2,4-oxadiazole moiety led to a series of derivatives which showed good antiproliferative activity in pancreatic cancer cell lines, associated with glycogen synthase kinase 3 β (GSK3-β) or CDK1 inhibition [27,28].

**Figure 2:**
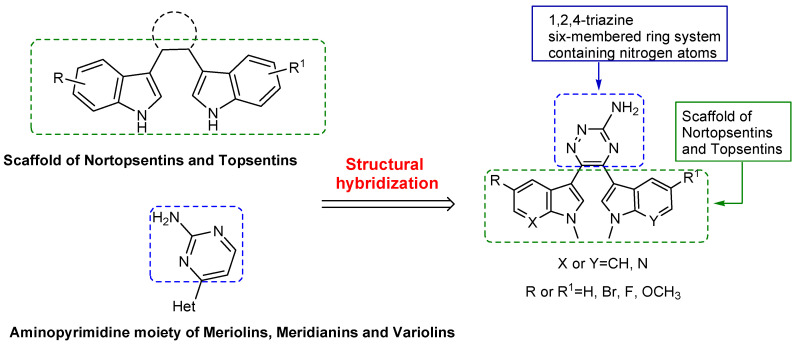
Drug design of azaindole triazine marine analogs using a structural hybridization approach.

In order to identify novel agents for pancreatic cancer treatment targeting protein kinases involved in the cancer metabolism and still unexplored, we mainly focus our attention on reviewing the progress made in the development of PDKs inhibitors. Since for the latter, including dichloroacetate (DCA), AZD7545, radicicol and M77976, no specific chemical features responsible for the PDKs inhibition were recognized [4], we chose to adopt a molecular hybridization approach that combines different pharmacophoric sub-units in a single structure, to design the novel PDK inhibitors. Among the molecules isolated from natural sources, compounds bearing a six-membered ring system, such as the aminopyrimidine, showed potent kinases inhibition [19,20]. We decided to investigate the effect of the substitution of the aminopyrimidine scaffold with a 3-amino-1,2,4-triazine central core, which was widely described for its antitumor activity [29,30,31,32,33]. Meanwhile, the bioactive bis-indolyl scaffold of nortopsentin and topsentin was maintained, resulting in the novel 1,2,4-triazine-based marine analogs reported in Figure 2.

The antiproliferative activity of the novel triazine compounds was evaluated in pancreatic cancer cell lines, demonstrating their ability to hamper cancer cell growth. Moreover, in order to elucidate the mechanism of action and the ability of the new compounds to revert altered pancreatic cancer cells metabolism, a specific enzymatic assay against the four isoforms of PDKs was carried out. The results showed a selective inhibition of PDK1 and PDK4 isoforms, which was further confirmed by molecular docking analysis.

## 2. Results and Discussion

### 2.1. Synthesis

**Scheme 1:**
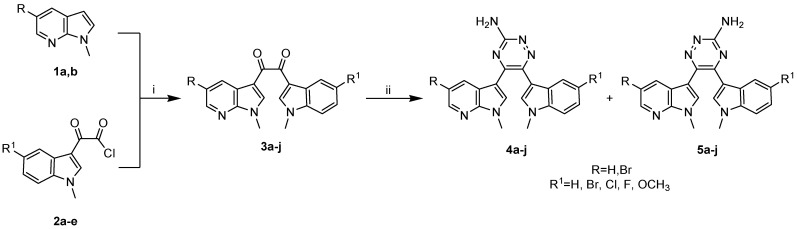
Synthesis of azaindole triazine derivatives **4a**–**j, 5a**–**j**. Reagents and conditions: (i) AlCl_3_, DCM/Heptane 2:1, RT, 2 h, 76–92%; (ii) aminoguanidine bicarbonate, *n*-butanol, reflux, 3 h, overall yields: 79–96%, **4a**–**j**: 40–49%, **5a**–**j**: 37–48%.

The synthesis of 1,2,4-triazines **4**–**5** was accomplished as depicted in Scheme 1. The key intermediate diketones **3a**–**j** were synthesized by Friedel–Crafts acylation of methylindole precursor of type **1a,b** with indolyl-oxo-acetyl chlorides **2a**–**e**, synthesized as previously described [28,34,35]. This reaction, performed in a mixture of DCM/heptane and in the presence of AlCl_3_ as a strong Lewis acid catalyst, was carried out via electrophilic aromatic substitution to form the 1,2- diones **3a**–**j** in good yields (76–92%).

The subsequent reaction of α-diketones **3a**–**j** with aminoguanidine bicarbonate produced new specimens of 3-amino-substituted 1,2,4-triazines **4a**–**j**, together with the corresponding structural isomers **5a**–**j**. All the synthesized derivatives **4a**–**j** and **5a**–**j** and their relative yields are summarized in Table 1.

### 2.2. Enzyme Inhibition Activity

**Figure 3:**
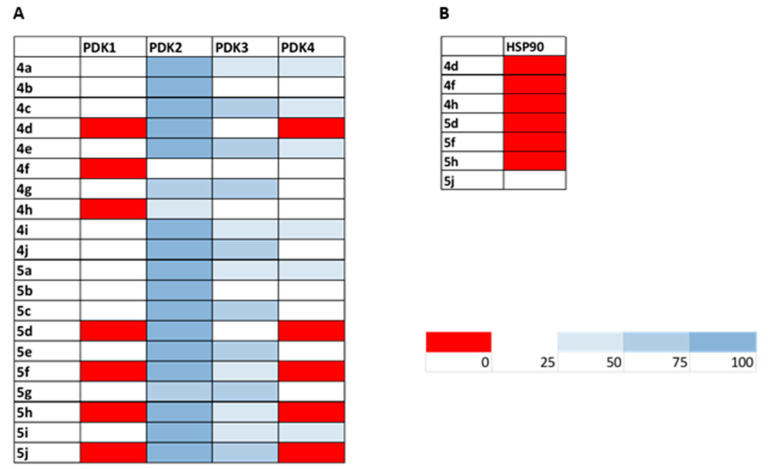
Inhibition of isolated enzyme isoforms. Heat-map showing the relative inhibition of tested compounds (at 1.5 µM) on PDK1-4 (**panel A**) and HSP90 (**panel B**) enzymes. Results are expressed as enzyme residual activity (range 0–100%). Data are the means of three independent experiments.

The ability of the tested compounds to hamper the activity of PDK1-4 isoforms was firstly evaluated by means of a chemioluminescent assay, the ADPGlo™ Kinase Assay kit (Promega Corporation, Madison, WI, USA). The results obtained by incubating PDK1-4 isoforms for 30 min at room temperature with 1.5 µM of tested compounds are depicted in Figure 3 panel A as a heat-map summary of the inhibition percentage on PDK1-4 enzymatic activity. All the newly synthesized compounds were able to inhibit the pyruvate dehydrogenase kinases, although to a different extent and showing a preferential activity towards the PDK1 and PDK4 isoforms. In particular, compounds **4d**, **4f**, **4h**, **5d**, **5f**, **5h** and **5j** totally hampered the enzymatic activity of PDK1 at 1.5 µM. Derivatives **4d**, **5d**, **5f**, **5h** and **5j** were also able to completely suppress the PDK4 activity. Conversely, all tested 1,2,4-triazin-3-amines were barely effective in blocking PDK2 activity at 1.5 µM concentration. In addition, IC_50_ values on the PDK1 isoform were also calculated for all synthesized derivatives and the results are shown in Table 2. All compounds were extremely efficient in decreasing PDK1 catalytic activity at sub-micromolar/nanomolar concentrations, with IC_50_ values ranging from 0.04 to 0.33 µM. Noticeably, all 1,2,4-triazin-3-amines prominently outperformed the well-known PDK reference inhibitors, DAP and DCA. 

Since the ATP binding pockets in PDKs and HSP90 are similar and taking into consideration that these 1,2,4-triazine derivatives conserved a chemical scaffold resembling that of the well-known Hsp90 inhibitor Ganetespib, the most performant PDK inhibitors **4d**, **4f**, **4h**, **5d**, **5f**, **5h** and **5j** were also screened for their ability to target HSP90. As reported in Figure 3 panel B, derivatives **4d**, **4f**, **4h**, **5d**, **5f**, and **5h** were highly effective against HSP90, being able to completely abolish the enzyme activity at 1.5 µM. Conversely, derivative **5j** was able to decrease HSP90 catalytic activity by only 60–80% at equi-doses, thus attesting to its ability to act as a more selective PDK1 inhibitor. While derivatives **4d**, **4f**, **4h**, **5d**, **5f**, and **5h** retained a similar inhibitory profile against HSP90, compound **5j** showed an IC_50_ value about two times higher towards isolated HSP90 compared with PDK-1 (Table 2).

### 2.3. Molecular Modeling Studies

The most appropriate protein–ligand complex of both HSP90 and PDK1 was selected on the basis of their crystallographic resolution, paying particular attention to the structures in which the co-crystallized ligand showed the highest structural similarity to compounds **4** and **5**. The crystal structures with PDB codes 2Q8F (method: X-ray diffraction, resolution: 2.03 Å) [36] and 5J64 (method: X-ray diffraction, resolution: 1.38 Å) [37] were chosen for PDK1 and HSP90, respectively. The PDB files were prepared for the computational analysis using MOE suite; first, the small missing loops in the structures were rebuilt trough the Structure Preparation program, then the proper protonation state was conferred employing Protonate 3D application, and finally, the added hydrogen atoms were minimized with the AMBER10:EHT [38] force field implemented in MOE.

The ligands, as well as the protein structures, underwent a proper preparation procedure, exploiting the tools of the QUACPAC package of the OpenEye suite. First, the tautomers tool was used for selecting the dominant tautomeric state for each compound, then the three-dimensional conformations for each molecule were generated with the OMEGA program, and the MolCharge tool was used to assign the proper partial charges (with the AM1BCC [39] method). Finally, the FixpKa application was exploited to determine the dominant protonation state for each ligand at pH 7.4.

For both HSP90 and PDK1, the ligands were docked in the orthosteric ATP-binding site with the program PLANTS which is based on an Ant-Colony-Optimization algorithm, which was developed by the University of Tübingen [40,41,42]. For each compound, 50 poses were generated. All the final conformations presenting steric clashes with the receptor were discarded, as well as the ones showing unfavorable electrostatic interactions within the binding pocket. In the case of HSP90, the remaining poses were filtered by passing them through a three-dimensional pharmacophoric model created by consensus on the different orthosteric HSP90 crystallographic ligand binding modes. For each of the remaining compounds, the final poses were inspected and the best one was selected.

**Figure 4:**
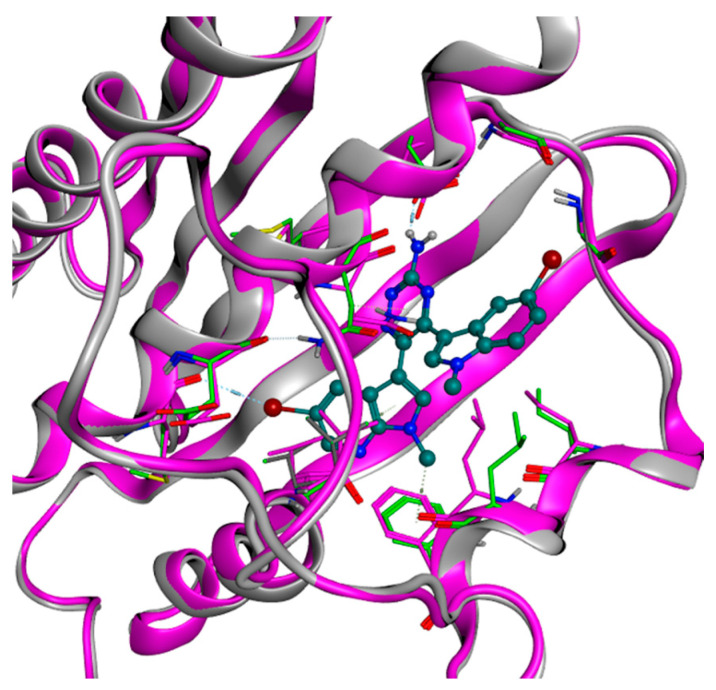
Representation of the superimposition between the original structure of PDK1 (PDB code: 2Q8F, colored in magenta) and our ligand-based homology model (depicted in grey), created on the manually placed conformation of compound **5d** (pine green in the figure). As can be seen, the side chains of the amino acids in the binding pocket have adapted to the presence of the ligand, while the backbone structure did not show significant changes (RMSD between the crystal and the model backbones: 0.87 Å). The shifted side chains of interest in the homology model have been highlighted in lime color. The image was created and rendered with MOE.

In the case of PDK1, molecular docking was not able to generate reasonable binding modes for the tested molecules, which displayed important clashes with the binding pocket. Knowing that the binding of these compounds is experimentally validated, we employed a technique called ligand-based homology modeling [43]. This method consists of the execution of a homology modeling operation, but with a defined ligand placed in a desired location (the ATP-binding site, in our case). The sequence to model is the same as the receptor of interest, indeed the main focus of the technique is to “reorganize” the amino acid side chain around the ligand, allowing it to get a reasonable placement in the pocket. All the ligand-based homology modeling passages were executed in MOE. The ligand chosen as the reference for the modeling was compound **5d**, one of the most potent derivatives of the series examined with respect to PDK1. Specifically, this compound was manually placed in the binding site, following our structure-activity relationship (SAR) hypothesis, which was also already reported in a previous study [43]. In this model, the triazine binds the residue Asp318, while one of the two bicyclic aromatic moieties is placed in the inner part of the pocket, leaving the other bicycle more exposed to the solvent. Figure 4 shows the clean superimposition of the protein backbones of the crystal structure and the model of PDK1, in which the side chains have moved to create the proper room for the ligand placement.

**Figure 5:**
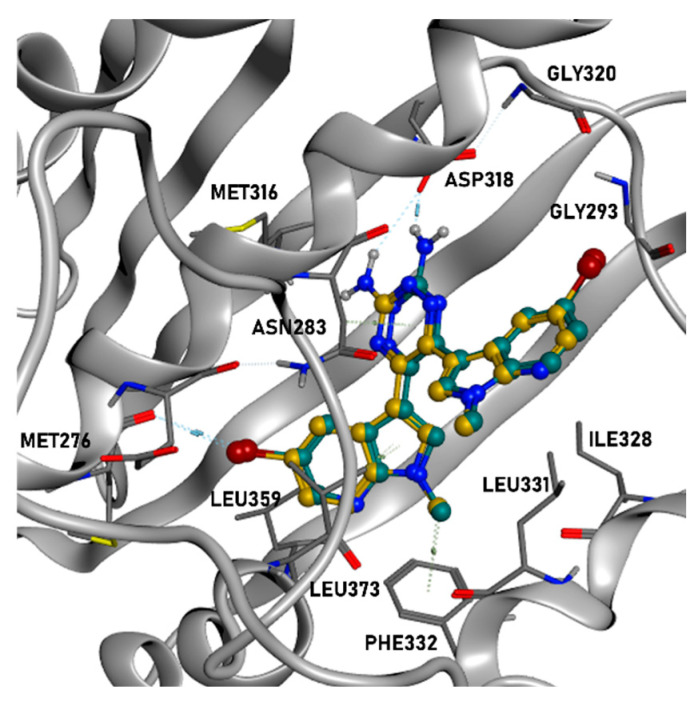
Image representing the best pose obtained for **5d** (highlighted in gold) and the originally manually placed conformation (colored in pine green), upon which the creation of the ligand-based homology model was run. As can be seen, the molecule orientation in the pocket is shifted of 180 degrees, but the interactive pattern is maintained. The image was created and rendered with MOE.

The molecular docking calculation was executed again on PDK1 using the model created instead of the crystallographic structure. The results were much more promising, with many clash-free conformations which also displayed a proficient electrostatic interaction with the binding site. It is important to highlight that the created poses for the ligands resemble the three-dimensional placement of compound **5d** in the pocket, giving credit to our 3D SAR hypothesis (see Figure 5 as an example). Indeed, even if the best docking pose displays a 180 degrees rotation of the ligand through the longitudinal axis, the interaction pattern with the protein is retained. Lacking several PDK1-ligand complexes in public databases, the best conformation for each ligand was selected based on the main literature information about the interaction with this target. 

**Figure 6:**
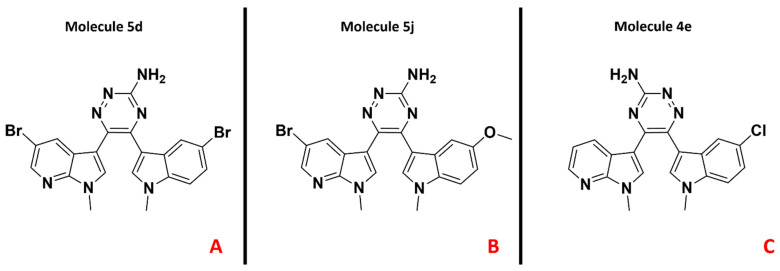
Representation of the chemical structures of the molecules taken as reference for the present study. Compound **5d** (PDK1 IC_50_= 0.05 µM, HSP90 IC_50_= 0.07 µM), **5j** (PDK1 IC_50_= 0.04 µM, HSP90 IC_50_= 0.18 µM), and **4e (**PDK1 IC_50_= 0.29 µM, not tested on HSP90) are depicted in (**panel A**), (**panel B**), and (**panel C**), respectively.

**Figure 7:**
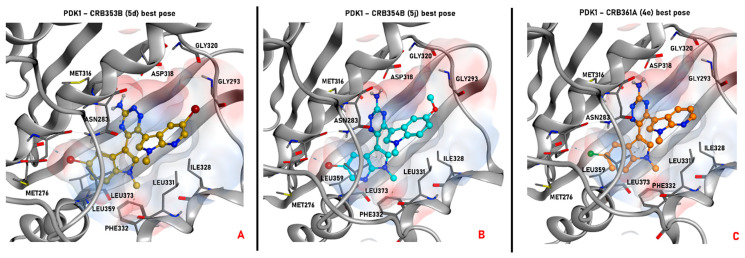
Representation of the selected pose produced by molecular docking with the program PLANTS for the compound **5d** (colored in gold, **panel A**), **5j** (depicted in cyan, **panel B**), and **4e** (colored in orange, **panel C**) in the ATP-binding site of the ligand-based homology model created for PDK1. All the selected conformations passed the steric and electrostatic filtering processes. In each panel, also the electrostatic surface around the ligand in the binding site is represented. The images were created and rendered with MOE.

**Figure 8:**
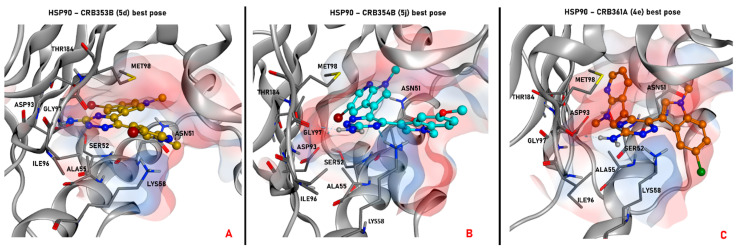
Representation of the selected pose produced by molecular docking with the program PLANTS for the compound **5d** (colored in gold, **panel A**), **5j** (depicted in cyan, **panel B**), and **4e** (colored in orange, **panel C**) in the ATP-binding site of HSP90. All the conformations passed the steric, electrostatic, and pharmacophoric filtering procedures. In each panel, also the electrostatic surface around the ligand in the binding site is represented. The images were created and rendered with MOE.

In order to confirm the experimental data presented in this study from a molecular modeling perspective, we decided to focus our analysis on three specific compounds. The first, compound **5d**, shows a very high-potency profile on both PDK1 (IC_50_ = 0.05 µM) and HSP90 (IC_50_ = 0.07 µM). The second, compound **5j**, has shown a significant inhibitory activity on PDK1 (IC_50_ = 0.04 µM) and a slightly lower potency against HSP90 (IC_50_ = 0.18 µM on HSP90), while the third, derivative **4e**, displays an even lower activity on PDK1 (IC_50_ = 0.29 µM), while it was not tested on HSP90. The selected ligands are represented in Figure 6, while their binding modes with the targets under examination are depicted in Figure 7 and Figure 8.

As depicted, all compounds interact through a hydrogen bond with Asp318 in the PDK1 ATP-binding site using their aromatic amine moiety. Moreover, all the molecules place the halogenated bicyclic portion in the inner part of the binding site, establishing a halogen bond with Met276. No significant differences between the binding poses for azaindole or indole insertion in the enzymatic pocket (both proficiently occy the volume of the region) were observed, as the only feature which seems to be relevant is the presence of a halogen atom (bromine or chlorine) for the stabilization of the cited molecular contact. Additionally, the methyl group of the indole (in the case of **5j**) and azaindole (for **5d** and **4e**) establishes a hydrophobic contact with the phenyl side chain of Phe332.

Focusing on the difference between the binding modes of **5d** and **5j**, it seems that the pocket occupancy and the halogen bond with Met276 play a more important role in ligand stabilization in respect to the hydrogen bond with Asp318. Moreover, the bromine seems to guide such interaction in a more proficient way in respect to chlorine, and this can be appreciated considering that **4e** retains the same interaction pattern of the other two examined ligands, but showing a 6-fold decrease in potency. Nevertheless, looking at the differences in potencies between the regioisomers of series **4** and **5**, most probably the hydrogen bond with Asp318 “guides” the binding event, favoring the placement of the ligand with an halogen directed towards Met276, proficiently occupying the pocket. This can be hypothesized taking into account that regioisomers with the same substitions in R and R_1_ (e.g, **4a** vs. **5a**, **4d** vs. **5d**) do not show noticeable differences in potencies, while there are some cases in which, when R and R_1_ are different (e.g., **4b** vs. **5b**, **4g** vs. **5g**, **4j** vs. **5j**), there is the tendency for a shift in favor to one of the two isomers. The case of **4j** vs. **5j** is representative because, being the compounds characterized by the bulky and rotatable methoxy substituent, it could be important for a binding perspective to place this moiety in the outer part of the pocket, and this event could be “guided” more proficiently by H-bonding with Asp318 for 5j in respect to **4j**. 

A slightly different situation is retrievable with HSP90. Indeed, while the binding modes of compounds **5j** and **4e** are very similar to each other, with the aromatic amine in contact with Gly97 and the azaindole in the inner portion of the pocket, the conformational bound form for **5d** is much more “flat”, with the ligand core directed to the left part of the ATP-binding site (the hydrogen bond is established with Asp93). 

Differently from what was observed for the previously described series of analogues [44,45], the potencies of compounds **4** and **5** are similar for both PDK1 and HSP90. 

Most probably, the key differences in the activity of these molecules are due to binding or unbinding kinetics rather than their interaction pattern in the pocket. Indeed, it is very likely that some dynamic processes affect the way in which the compounds approach the binding site, allowing some molecules to enter and place in the pocket more easily than others. This kind of behavior can be analyzed with molecular dynamics (MD) simulations, and more specifically with methodologies able to sample the protein–ligand recognition pathway. One of the techniques which have shown success in this field is Supervised Molecular Dynamics (SuMD) [46], an unbiased enhanced sampling approach able to proficiently describe the recognition event between a ligand and a biological target in the nanosecond timescale. This method has been already applied to several targets and scenarios, giving very promising results [47,48,49,50] 

On the other side, if the main discriminant in the potency discrepancy is related to the unbinding process, some other MD methodologies can be applied. This is the case for some enhanced sampling methods, such as steered MD [51] or Thermal Titration Molecular Dynamics (TTMD) [52,53]. Specifically, this last technique has been recently developed, and its main advantage is that it is not based on the introduction of energetical bias in the system. Indeed, the system temperature is the only parameter that is changed in the simulation in order to allow for more proficient discrimination of the ligands based on their residence time, which is progressively increased with simulation time, in the binding pocket. 

Currently, a computational work combining SuMD and TTMD is taking place in our laboratory, with the specific goal to rationalize the potency discrepancies in this and other series of ligands, with a specific focus on PDK1 and HSP90. Indeed, the very high similarities of these proteins, in particular in their ATP-binding site [44], suggest that the binding/unbinding events are not extremely important for understanding potency discrepancies within the series of ligands considered. We are confident that the combination of SuMD and TTMD will help to elucidate this very impactful aspect, also allowing to guide further efforts in drug design of new PDK inhibitors. 

### 2.4. 2D and 3D Cytotoxicity Studies

All newly developed 1,2,4-amino-triazine compounds were screened for their cytotoxic activity against two human PDAC cell lines, namely, a KRAS-wild-type PDAC cell line (BxPC-3 cells) and a KRAS-mutant PDAC cell line (PSN-1 cells). For comparison purposes, the efficacy of the PDAC gold-standard chemotherapeutic gemcitabine, as well as of the well-known PDK inhibitors DCA and DAP were assessed under the same experimental conditions. The cytotoxicity parameters, expressed in terms of IC_50_ and obtained after 72 h of drug exposure by MTT assay, are reported in Table 3.

As expected, DCA was effective at millimolar concentrations in determining a reduction in cancer cell viability. On the contrary, all tested compounds showed a promising cytotoxic activity, with IC_50_ values in the micromolar range. Remarkably, all derivatives were much more effective against KRAS mutant PSN1 cancer cells compared with wild-type BxPC-3 pancreatic cancer cells. On the other hand, all derivatives were less cytotoxic compared with the reference chemotherapeutic drug gemcitabine and some compounds were also less effective than DAP in decreasing cancer cell proliferation. Among all, compounds **4c**, **4g**, **5b** and **5j** were the most effective, being able to inhibit cell growth more potently than DAP. In particular, **5j** was extremely potent towards both considered PDAC cells, eliciting IC_50_ values in the sub-micromolar range.

Prompted by these encouraging results, we also screened the newly developed 1,2,4-triazine compounds against 3D spheroids of PDAC KRAS wild-type and mutant cells, to further estimate their anticancer potential in a more predictive environment. Actually, 3D cell cultures possess several features that closely resembles the in vivo tumor architecture complexity and pathophysiology, being more predictive for in vivo effectiveness with respect to bidimensional cancer cell models [54]. In particular, 3D spheroids are significantly more representative of the in vivo tumor metabolic conditions [55], thus emphasizing the need to use 3D cell models for investigating new putative drugs acting on tumor targets implicated in cancer metabolism, such as PDKs. The PDAC cancer spheroids were treated with tested compounds for 72 h, and cell viability was assessed by means of the acid phosphatase (APH) assay (Table 3). The efficacy of gemcitabine as well as of DCA and DAP was assessed under the same experimental conditions. Tested on 3D models, all compounds were much more effective than DCA, whereas only few derivatives (**4c**, **4g**, and **5b**) were more active than DAP. Interestingly, some derivatives showed an in vitro antitumor profile greater than gemcitabine on 3D pancreatic models. As for 2D PDAC models, all 1,2,4-triazine compounds were, on average, much more effective against KRAS mutant PSN-1 spheroids compared with KRAS wild-type. 3D assays confirmed derivatives **4c**, **4g**, **5b** and **5j** as the most effective, with IC_50_ values resembling those detected with the well-known PDK inhibitor DAP. 

## 3. Materials and Methods

### 3.1. Chemistry

The anhydrous solvents used for organic synthesis (*n-*butanol and heptane) and the reagents were purchased from Sigma-Aldrich Co, Alfa Aesar, VWR International, and Acros Organics. Dichloromethane was purified and dried using calcium hydride and stored over 4 Å molecular sieves. All air- or moisture-sensitive reactions were performed using oven-dried glassware under an inert dry nitrogen atmosphere. Analytical thin-layer chromatography (TLC) was performed on silica gel 60 F254 plates (0.25 mm thickness) and the developed plates were examined under ultraviolet (UV) light. All melting points were taken on a Buchi-Tottoly capillary apparatus and were uncorrected. IR spectra were determined in bromoform with a Shimadzu FT/IR 8400S spectrophotometer and peaks were reported in wavenumber (cm^−1^). ^1^H and ^13^C NMR spectra were measured at 200 and 50 MHz, respectively, on DMSO-*d_6_* solution, using a Bruker Avance II series 200 MHz spectrometer. Chemical shifts were described in parts per million (δ), coupling constants (J) were expressed in Hertz (Hz), and splitting patterns were reported as singlet (s), doublet (d), triplet (t), quartet (q), multiplet (m), doublet of doublets (dd) and triplet of doublets (td). The chromatography column was performed with MERK silica gel 230–400 mesh ASTM or FLASH40i Biotage chromatography or with Buchi Sepacore chromatography module (prepacked cartridge reference). We state that all compounds are >95% pure by LC-MS analysis. MS spectra of all final synthesized compounds are reported in the Supporting Information (Figures S1–S20). The LC–MS was performed using an Agilent 1260 Infinity system (Agilent Technologies, Waldbronn, Germany), quaternary pump and autosampler in association with a Varian MS 500 Ion Trap Mass Spectrometer (Agilent Technologies, Waldbronn, Germany). The analysis was performed using an eclipse plus C18, 4.6 × 150 mm column using the following gradient: 0.0 min 80% A, 20% B; 10 min 20% A, 80% B, 14 min 20% A, 80% B, 15 min 80% A, 20% A (A: MilliQ water 1% formic acid, B: acetonitrile). The analysis lasted 15 min with a 0.750 mL/min flow. The compounds were detected by electrospray ionization ion trap mass spectrometry source under positive-ion conditions in turbo TDDS in the acquisition range *m/z* 300–550. The following parameters were used: capillary voltage 95.0 V, Needle Voltage +/− 5500 V, RF loading 81%. 

#### 3.1.1. General procedure for the preparation of 1-(1-Methyl-1H-indol-3-yl)-2-(1-methyl-1H-pyrrolo [2,3-b]pyridin-3-yl)-ethane-1,2-dione (3**a**–**j**)

To a suspension of acyl chloride **2a**–**e** (2.41 mmol) in a mixture of DCM/heptane 2:1 (7.2 mL), under a nitrogen atmosphere, aluminum trichloride (10.84 mmol) was added in small portions. The reaction mixture was left to stir at room temperature for a few minutes and then a solution of the proper 7-azaindole **1a,b** (3.98 mmol) in DCM (2.4 mL) was added dropwise. When the reaction reached completion, monitored by TLC, the crude mixture was poured into crushed ice, until the destruction of the aluminum trichloride, which is manifested by the cessation of effervescence. The resulting solid precipitate was collected by vacuum filtration, washed with water, dried under vacuum for 24 h, and purified by column chromatography using DCM/ethyl acetate 7:3 as eluent to give the desired products **3a**–**j**.

1-(1-Methyl-1*H*-indol-3-yl)-2-(1-methyl-1*H*-pyrrolo[2,3-*b*]pyridin-3-yl)-ethane-1,2-dione **(3a)** Yield: 78%; light yellow solid; m.p.: 242.4 °C; IR (cm^−1^): 1697 (CO), 1601 (CO); ^1^H NMR (DMSO-*d_6,_* 200 MHz) δ: 8.57 (dd, *J* = 7.8, 1.6 Hz), 8.52 (s, 1H), 8.45 (dd, *J* = 4.7, 1.6 Hz, 1H), 8.32 (s, 1H), 8.31–8.29 (m, 1H), 7.62–7.59 (m, 1H), 7.40–7.32 (m, 4H), 3.98 (s, 3H), 3.90 (s, 3H); ^13^C{^1^H} NMR (DMSO-*d_6_*, 50 MHz) δ: 188.6; 187.9, 148.7, 145.1, 141.5, 141.4, 138.0, 130.3, 126.5, 124.1, 123.4, 121.9, 119.4, 118.9, 111.6, 111.5, 110.3, 33.8, 32.1.

1-(5-Bromo-1-methyl-1*H*-pyrrolo[2,3-*b*]pyridin-3-yl)-2-(1-methyl-1*H*-indol-3-yl)-ethane-1,2-dione **(3b)** Yield: 83%; ochre solid; m.p.: 217.0 °C; IR (cm^−1^): 1620 (CO), 1616 (CO); ^1^H NMR (DMSO-*d_6,_* 200 MHz) δ: 8.68 (d, *J* = 2.3 Hz, 1H), 8.60 (s, 1H), 8.54 (d, *J* = 2.3 Hz, 1H), 8.35 (s, 1H), 8.30–8.28 (m, 1H), 7.62–7.60 (m, 1H), 7.40–7.32 (m, 2H), 3.89 (s, 3H), 3.88 (s, 3H); ^13^C{^1^H} NMR (DMSO-*d_6_*, 50 MHz) δ: 188.2, 187.2, 147.2, 145.2, 142.6, 141.7, 138.0, 132.0, 126.5, 124.1, 123.5, 121.9, 120.5, 114.9, 111.6, 111.5, 109.7, 33.8, 32.4.

1-(5-Bromo-1-methyl-1*H*-indol-3-yl)-2-(1-methyl-1*H*-pyrrolo[2,3-*b*]pyridin-3-yl)-ethane-1,2-dione **(3c)** Yield: 90%; light yellow solid; m.p.: 278.1 °C; IR (cm^−1^): 1625 (CO), 1605 (CO); ^1^H NMR (DMSO-*d_6,_* 200 MHz) δ: 8.57 (d, *J* = 1.5 Hz, 1H), 8.55 (s, 1H), 8.46–8.44 (m, 2H), 8.39 (s, 1H), 7.62 (d, *J* = 8.7 Hz, 1H), 7.52 (dd *J* = 8.7, 2.0 Hz, 1H), 7.40 (dd, *J* = 7.9, 4.7 Hz, 1H), 3.90 (s, 3H), 3.89 (s, 3H); ^13^C{^1^H} NMR (DMSO-*d_6_*, 50 MHz) δ: 188.0; 187.6, 148.7, 145.1, 142.4, 141.6, 136.8, 130.3, 128.3, 126.6, 124.1, 119.5, 118.9, 116.3, 113.8, 111.1, 110.1, 34.0, 32.2. 

1-(5-Bromo-1-methyl-1*H*-indol-3-yl)-2-(5-bromo-1-methyl-1*H*-pyrrolo[2,3-*b*]pyridin-3-yl)-ethane-1,2-dione **(3d)** Yield: 86%; cream solid; m.p.: 287.6 °C; IR (cm^−1^): 1624 (CO), 1616 (CO); ^1^H NMR (DMSO-*d_6,_* 200 MHz) δ: 8.68 (d, *J* = 2.2 Hz, 1H), 8.63 (s, 1H), 8.55 (d, *J* = 2.2 Hz, 1H), 8.43 (d, *J* = 2.0 Hz, 1H), 8.42 (s, 1H), 7.62 (d, *J* = 8.7 Hz, 1H), 7.53 (d, *J* = 2.0 Hz, 1H), 3.89 (s, 6H); ^13^C{^1^H} NMR (DMSO-*d_6_*, 50 MHz) δ: 187.6, 187.0, 147.2, 145.3, 142.9, 142.6, 136.8, 132.0, 128.3, 126.7, 124.0, 120.5, 116.4, 114.9, 113.9, 110.9, 109.6, 34.0, 32.4.

1-(5-Chloro-1-methyl-1*H*-indol-3-yl)-2-(1-methyl-1*H*-pyrrolo[2,3-*b*]pyridin-3-yl)-ethane-1,2-dione **(3e)** Yield: 89%; wheat solid; m.p.: 263.0 °C; IR (cm^−1^): 1624 (CO), 1616 (CO); ^1^H NMR (DMSO-*d_6,_* 200 MHz) δ: 8.56 (dd, *J* = 7.8, 1.6 Hz, 1H), 8.55 (s, 1H), 8.45 (dd, *J* = 4.7, 1.6 Hz, 1H), 8.41 (s, 1H), 8.28 (d, *J* = 2.1 Hz, 1H), 7.68– 7.66 (m, 1H), 7.41- 7.38 (m, 2H), 3.90 (s, 3H), 3.89 (s, 3H); ^13^C{^1^H} NMR (DMSO-*d_6_*, 50 MHz) δ: 188.0, 187.6, 148.7, 145.1, 142.5, 141.6, 136.5, 130.3, 128.2, 127.7, 124.0, 121.0, 119.5, 118.9, 113.4, 111.2, 110.1, 34.0, 32.1.

1-(5-Bromo-1-methyl-1*H*-pyrrolo[2,3-*b*]pyridin-3-yl)-2-(5-chloro-1-methyl-1*H*-indol-3-yl)-ethane-1,2-dione **(3f)** Yield: 76%; light yellow solid; m.p.: 271.0 °C; IR (cm^−1^): 1643 (CO), 1630 (CO); ^1^H NMR (DMSO-*d_6,_* 200 MHz) δ: 8.68 (d, *J* = 2.2 Hz, 1H); 8.63 (s, 1H), 8.55 (d, *J* = 2.2 Hz, 1H), 8.43 (s, 1H), 8.27 (d, *J* = 2.1 Hz, 1H), 7.67 (d, *J* = 8.7 Hz, 1H), 7.40 (dd, *J* = 8.7, 2.1 Hz, 1H), 3.90 (s, 3H), 3.89 (s, 3H); ^13^C{^1^H} NMR (DMSO-*d_6_*, 50 MHz) δ: 187.6, 187.0, 145.3, 142.8, 142.7, 136.5, 132.1, 128.4, 128.3, 128.2, 124.1, 121.0, 120.5, 114.9, 113.5, 111.0, 109.6, 34.1, 32.4. 

1-(5-Fluoro-1-methyl-1*H*-indol-3-yl)-2-(1-methyl-1*H*-pyrrolo[2,3-*b*]pyridin-3-yl)-ethane-1,2-dione **(3g)** Yield: 91%; ochre solid; m.p.: 238.6 °C; IR (cm^−1^): 1628 (CO), 1616 (CO); ^1^H NMR (DMSO-*d_6,_* 200 MHz) δ: 8.57 (dd, *J* = 7.8, 1.6 Hz, 1H), 8.55 (s, 1H), 8.45 (dd, *J* = 4.7, 1.6 Hz, 1H), 8.40 (s, 1H), 7.96 (dd, *J* = 9.6, 2.6 Hz, 1H), 7.65 (dd, *J* = 9.0, 4.4 Hz, 1H), 7.39 (dd, *J* = 7.8, 4.7 Hz, 1H), 7.23 (td, *J* = 9.2, 9.0, 2.6 Hz, 1H), 3.90 (s, 3H), 3.89 (s, 3H); ^13^C{^1^H} NMR (DMSO-*d_6_*, 50 MHz) δ: 188.1, 187.6, 159.8 (d, *J*= 236.0.3 Hz), 148.7, 145.1, 142.7, 141.5, 134.6, 130.3, 127.3 (d, *J* = 11.4 Hz), 119.5, 118.9, 113.1 (d, *J*= 9.9 Hz), 112.1 (d, *J* = 26.0 Hz), 111.5 (d, *J* = 3.8 Hz), 110.1, 106.8 (d, *J* = 25.1 Hz), 34.1, 32.1. 

1-(5-Bromo-1-methyl-1*H*-pyrrolo[2,3-*b*]pyridin-3-yl)-2-(5-fluoro-1-methyl-1*H*-indol-3-yl)-ethane-1,2-dione **(3h)** Yield: 87%; yellow solid; m.p.: 249.7 °C; IR (cm^−1^): 1636 (CO), 1611 (CO); ^1^H NMR (DMSO-*d_6,_* 200 MHz) δ: 8.68 (d, *J* = 2.2 Hz, 1H), 8.62 (s, 1H), 8.54 (d, *J* = 2.2 Hz, 1H), 8.43 (s, 1H), 7.95 (dd, *J* = 9.6, 2.6 Hz, 1H), 7.66 (dd, *J* = 9.0, 4.4 Hz, 1H), 7.23 (td, *J* = 9.2, 9.0, 2.6 Hz, 1H), 3.89 (s, 3H), 3.88 (s, 3H); ^13^C{^1^H} NMR (DMSO-*d_6_*, 50 MHz) δ: 187.8, 186.9, 159.8 (d, *J* = 236.0.3 Hz), 147.2, 145.3, 142.9, 142.7, 134.6, 132.0, 127.3 (d, *J* = 11.1 Hz), 120.5, 114.9, 113.2 (d*_,_ J* = 9.5 Hz), 112.1 (d, *J*= 25.8 Hz), 111.4 (d, *J* = 3.8 Hz), 109.6, 106.8 (d, *J*= 25.1 Hz), 34.1, 32.4.

1-(5-Methoxy-1-methyl-1*H*-indol-3-yl)-2-(1-methyl-1*H*-pyrrolo[2,3-*b*]pyridin-3-yl)-ethane-1,2-dione **(3i)** Yield: 84%; light yellow solid; m.p.: 204.8 °C; IR (cm^−1^): 1636 (NH), 1616 (CO); ^1^H NMR (DMSO-*d_6,_* 200 MHz) δ: 8.57 (dd, *J* = 7.8, 1.6 Hz, 1H), 8.54 (s, 1H), 8.45 (dd, *J* = 4.7, 1.6 Hz, 1H), 8.26 (s, 1H), 7.81 (d, *J* = 2.5 Hz, 1H), 7.51 (d, *J* = 8.9 Hz, 1H), 7.39 (dd, *J* = 7.8, 4.7 Hz, 1H), 6.98 (dd, *J* = 8.9, 2.5 Hz, 1H), 3.90 (s, 3H), 3.85 (s, 3H), 3.84 (s, 3H); ^13^C{^1^H} NMR (DMSO-*d_6_*, 50 MHz) δ: 188.6, 187.7, 156.8, 148.7, 145.1, 141.4, 141.3, 132.8, 130.3, 127.5, 119.4, 118.9, 113.6, 112.5, 111.3, 110.3, 103.8, 55.8, 33.9, 32.1. 

1-(5-Bromo-1-methyl-1*H*-pyrrolo[2,3-*b*]pyridin-3-yl)-2-(5-methoxy-1-methyl-1*H*-indol-3-yl)-ethane-1,2-dione **(3j)** Yield: 92%; ochre solid; m.p.: 264.6 °C; IR (cm^−1^): 1636 (CO), 1612 (CO); ^1^H NMR (DMSO-*d_6,_* 200 MHz) δ: 8.71–8.56 (m, 3H), 8.36–8.24 (m, 1H), 7.89–7.78 (m, 1H), 7.60–7.48 (m, 1H), 7.03–7.00 (m, 1H), 3.91–3.80 (m, 9H); ^13^C{^1^H} NMR (DMSO-*d_6_*, 50 MHz) δ: 187.0, 186.9, 156.9, 147.9, 145.3, 142.6, 141.6, 132.9, 132.1, 127.5, 120.7, 114.8, 113.7, 112.6, 111.2, 103.8, 55.9, 34.0, 32.4.

#### 3.1.2. General procedure for the preparation of 5,6-di(1H-indol-3-yl)-1,2,4-triazin-3-amines **(4**–**5)**

To a suspension of the appropriate derivatives **3a**–**j** (5 mmol) in anhydrous *n*- butanol (20 mL), aminoguanidine bicarbonate (10 mmol) was added in portions. The resulting mixture was heated under reflux for 3 h. The precipitate, which formed upon cooling, was collected by vacuum filtration, washed with *n*-butanol, and dried under vacuum for 24 h. The crude residue, obtained as a mixture of two diastereomers, was purified by column chromatography (CC) using dichloromethane/methanol 97:3 and 96:4 as eluent, affording the single enantiomers **4** and **5**, respectively. 

6-(1-Methyl-1*H*-indol-3-yl)-5-(1-methyl-1*H*-pyrrolo[2,3-*b*]pyridin-3-yl)-1,2,4-triazin-3-amine **(4a)** Yield: 42%; yellow solid; m.p.: 268.1 °C; IR (cm^−1^): 3458, 3271 (NH_2_); ^1^H NMR (DMSO-*d*_6,_ 200 MHz) δ: 8.81 (dd, *J* = 8.0, 1.6 Hz, 1H), 8.32 (dd, *J* = 4.7, 1.6 Hz, 1H), 7.63 (s, 1H), 7.54 (d, *J* = 8.2 Hz, 1H), 7.24 (s, 1H), 7.22- 7.16 (m, 3H), 6.99- 6.93 (m, 3H), 3.88 (s, 3H), 3.55 (s, 3H); ^13^C{^1^H} NMR (DMSO-*d_6_*, 50 MHz) δ: 161.3, 152.5, 148.1, 144.1, 143.4, 137.2, 134.5, 131.9, 130.1, 126.3, 122.0, 120.4, 119.9, 119.6, 117.6, 111.6, 110.7, 108.8, 33.1, 31.7; LC-MS: *m/z* [M + H]^+^ calcd for C_20_H_18_N_7_^+^ 356.40; found, 356.41.

5-(1-Methyl-1*H*-indol-3-yl)-6-(1-methyl-1*H*-pyrrolo[2,3-*b*]pyridin-3-yl)-1,2,4-triazin-3-amine **(5a)** Yield: 38%; yellow solid; m.p.: 235.5 °C; IR (cm^−1^): 3397, 3298 (NH_2_); ^1^H NMR (DMSO-*d*_6,_ 200 MHz) δ: 8.46 (d, *J* = 7.9 Hz, 1H) 8.28 (dd, *J* = 4.6, 1.6 Hz, 1H), 7.80 (s, 1H), 7.59 (dd, *J* = 7.9, 1.6 Hz, 1H), 7.44 (d, *J* = 8.1 Hz, 1H), 7.28–7.12 (m, 3H), 6.99 (dd, *J* = 7.9, 4.6 Hz, 1H), 6.95 (s, 2H), 3.88 (s, 3H), 3.56 (s, 3H); ^13^C{^1^H} NMR (DMSO-*d_6_*, 50 MHz) δ: 161.3, 153.1, 148.0, 143.2, 143.1, 137.4, 134.5, 130.1, 128.9, 127.1, 123.3, 122.9, 121.4, 118.6, 116.2, 110.8, 110.6, 110.0, 33.3, 31.5; LC-MS: *m/z* [M + H]^+^ calcd for C_20_H_18_N_7_^+^ 356.40; found, 356.38.

5-(5-Bromo-1-methyl-1*H*-pyrrolo[2,3-*b*]pyridin-3-yl)-6-(1-methyl-1*H*-indol-3-yl)-1,2,4-triazin-3-amine **(4b)** Yield: 44%; yellow solid; m.p.: 260.0 °C; IR (cm^−1^): 3466, 3447 (NH_2_); ^1^H NMR (DMSO-*d*_6,_ 200 MHz) δ: 8.92 (d, *J* = 2.2 Hz, 1H), 8.39 (d, *J* = 2.2 Hz, 1H), 7.65 (s, 1H), 7.54 (d, *J* = 8.2 Hz, 1H), 7.29 (s, 1H), 7.20–7.16 (m, 2H), 7.10 (s, 2H), 6.96- 6.92 (m, 1H), 3.89 (s, 3H), 3.53 (s, 3H); ^13^C{^1^H} NMR (DMSO-*d_6_*, 50 MHz) δ: 161.3, 151.9, 146.5, 144.2, 143.2, 137.2, 135.9, 133.6, 130.1, 126.2, 122.0, 121.1, 120.3, 120.0, 113.4, 111.4, 110.7, 108.5, 33.1, 31.9; LC-MS: *m/z* [M + H]^+^ calcd for C_20_H_17_BrN_7_^+^ 434.07; found, 436.30.

6-(5-Bromo-1-methyl-1*H*-pyrrolo[2,3-*b*]pyridin-3-yl)-5-(1-methyl-1*H*-indol-3-yl)-1,2,4-triazin-3-ylamine **(5b)** Yield: 42%; yellow solid; m.p.: 275.5.0 °C; IR (cm^−1^): 3472, 3447 (NH_2_); ^1^H NMR (DMSO-*d_6,_* 200 MHz) δ: 8.37–8.25 (m, 2H), 7.87 (d, *J* = 2.2 Hz, 1H), 7.82 (s, 1H), 7.47 (d, *J* = 8.2 Hz, 1H), 7.35 (s, 1H), 7.28–7.20 (m, 1H), 7.14 (t, *J* = 7.5 Hz, 1H), 6.99 (s, 2H), 3.84 (s, 3H), 3.64 (s, 3H); ^13^C{^1^H} NMR (DMSO-*d_6_*, 50 MHz) δ: 161.3, 146.5, 143.2, 142.8, 137.4, 137.2, 134.4, 131.9, 130.9, 127.0, 123.1, 122.9, 121.3, 120.5, 111.6, 110.8, 110.1, 110.0, 33.3, 31.7; LC-MS: *m/z* [M + H]^+^ calcd for C_20_H_17_BrN_7_^+^ 434.07; found, 434.42.

6-(5-Bromo-1-methyl-1*H*-indol-3-yl)-5-(1-methyl-1*H*-pyrrolo[2,3-*b*]pyridin-3-yl)-1,2,4-triazin-3-amine **(4c)** Yield: 49%; yellow solid; m.p.: 176.3 °C; IR (cm^−1^): 3481, 3402 (NH_2_); ^1^H NMR (DMSO-*d_6,_* 200 MHz) δ: 8.68 (dd, *J* = 8.0, 1.5 Hz, 1H), 8.33 (dd, *J* = 4.6, 1.4 Hz, 1H), 7.63 (s, 1H), 7.57–7.47 (m, 2H), 7.40 (s, 1H), 7.31 (dd, *J* = 8.7, 1.9 Hz, 1H), 7.19 (dd, *J* = 8.0, 4.7 Hz, 1H), 7.02 (s, 2H), 3.84 (s, 3H), 3.64 (s, 3H); ^13^C{^1^H} NMR (DMSO-*d_6_*, 50 MHz) δ: 161.1, 152.6, 144.1, 143.0, 136.0, 135.4, 134.4, 131.7, 131.6, 128.2, 124.5, 122.7, 119.5, 117.6, 112.8, 112.7, 111.0, 108.9, 33.3, 31.7; LC-MS: *m/z* [M + H]^+^ calcd for C_20_H_17_BrN_7_^+^ 434.07; found, 434.39.

5-(5-bromo-1-methyl-1*H*-indol-3-yl)-6-(1-methyl-1*H*-pyrrolo[2,3-*b*]pyridin-3-yl)-1,2,4-triazin-3-amine **(5c)** Yield: 45%; yellow solid; m.p.: 304.5 °C; IR (cm^−1^): 3304, 3127 (NH_2_); ^1^H NMR (DMSO-*d_6,_* 200 MHz) δ: 8.56 (d, *J* = 1.7 Hz, 1H), 8.28 (dd, *J* = 4.6, 1.2 Hz, 1H), 7.82 (s, 1H), 7.55 (dd, *J* = 7.9, 1.4 Hz, 1H), 7.43 (d, *J* = 8.7 Hz, 1H), 7.36 (dd, *J* = 8.7, 1.9 Hz, 1H), 7.24 (s, 1H), 7.05 (s, 2H), 6.98 (dd, *J* = 7.9, 4.6 Hz, 1H), 3.89 (s, 3H), 3.55 (s, 3H); ^13^C{^1^H} NMR (DMSO-*d_6_*, 50 MHz) δ: 161.3, 152.6, 148.0, 143.3, 136.8, 136.1, 135.6, 130.2, 128.8, 128.7, 125.5, 125.2, 118.5, 116.2, 114.6, 112.9, 110.4, 109.6, 33.5, 31.5; LC-MS: *m/z* [M + H]^+^ calcd for C_20_H_17_BrN_7_^+^ 434.07; found, 434.40.

6-(5-Bromo-1-methyl-1*H*-indol-3-yl)-5-(5-bromo-1-methyl-1*H*-pyrrolo[2,3-*b*]pyridin-3-yl)-1,2,4-triazin-3-amine **(4d)** Yield: 48%; yellow solid; m.p.: 266.5.0 °C; IR (cm^−1^): 3481, 3285 (NH_2_); ^1^H NMR (DMSO-*d_6,_* 200 MHz) δ: 8.46 (d, *J* = 1.9 Hz, 1H), 8.35 (d, *J* = 2.2 Hz, 1H), 7.84–7.83 (m, 2H), 7.46 (d, *J* = 8.7 Hz, 1H), 7.38–7.35 (m, 2H), 7.08 (s, 2H), 3.85 (s, 3H), 3.62 (s, 3H); ^13^C{^1^H} NMR (DMSO-*d_6_*, 50 MHz) δ: 161.3, 152.6, 146.5, 143.3, 142.7, 136.2, 135.5, 131.9, 130.8, 128.6, 125.5, 125.0, 120.4, 114.5, 112.9, 111.6, 110.0, 109.6, 33.5, 31.7; LC-MS: *m/z* [M + H]^+^ calcd for C_20_H_16_Br_2_N_7_^+^ 513.97; found, 514.30.

5-(5-Bromo-1-methyl-1*H*-indol-3-yl)-6-(5-bromo-1-methyl-1*H*-pyrrolo[2,3-*b*]pyridin-3-yl)-1,2,4-triazin-3-amine **(5d)** Yield: 44%; yellow solid; m.p.: 333.7 °C; IR (cm^−1^): 3426, 3315 (NH_2_); ^1^H NMR (DMSO-*d_6,_* 200 MHz) δ: 8.82 (d, *J* = 2.3 Hz, 1H), 8.40 (d, *J* = 2.2 Hz, 1H), 7.65 (s, 1H), 7.53 (d, *J* = 8.7 Hz, 1H), 7.48 (d, *J* = 1.8 Hz, 1H), 7.44 (s, 1H), 7.31 (dd, *J* = 8.7, 2.0 Hz, 1H), 7.13 (s, 2H), 3.85 (s, 3H), 3.62 (s, 3H); ^13^C{^1^H} NMR (DMSO-*d_6_*, 50 MHz) δ: 161.3, 152.0, 146.6, 144.3, 142.8, 136.0, 135.7, 133.4, 131.6, 128.2, 124.5, 122.6, 121.0, 113.3, 112.9, 112.8, 110.9, 108.5, 33.3, 31.9; LC-MS: *m/z* [M + H]^+^ calcd for C_20_H_16_Br_2_N_7_^+^ 513.97; found, 514.28.

6-(5-Chloro-1-methyl-1*H*-indol-3-yl)-5-(1-methyl-1*H*-pyrrolo[2,3-*b*]pyridin-3-yl)-1,2,4-triazin-3-amine **(4e)** Yield: 46%; yellow solid; m.p.: 178.0 °C; IR (cm^−1^): 3481, 3408 (NH_2_); ^1^H NMR (DMSO-*d*_6,_ 200 MHz) δ: 8.70 (dd, *J* = 8.0, 1.6 Hz, 1H), 8.33 (dd, *J* = 4.7, 1.6 Hz, 1H), 7.60–7.56 (m, 1H), 7.65 (s, 1H), 7.38 (s, 1H), 7.37–7.28 (m, 1H), 7.25–7.10 (m, 2H), 7.02 (s, 2H), 3.85 (s, 3H), 3.63 (s, 3H); ^13^C{^1^H} NMR (DMSO-*d_6_*, 50 MHz) δ: 161.3, 152.5, 148.2, 144.1, 143.0, 135.7, 134.3, 131.7 (×2), 127.6, 124.8, 122.0, 119.6, 119.5, 117.6, 112.4, 111.2, 108.9, 33.3, 31.7; LC-MS: *m/z* [M + H]^+^ calcd for C_20_H_17_ClN_7_^+^ 390.12; found, 390.40.

5-(5-Chloro-1-methyl-1*H*-indol-3-yl)-6-(1-methyl-1*H*-pyrrolo[2,3-*b*]pyridin-3-yl)-1,2,4-triazin-3-amine **(5e)** Yield: 41%; yellow solid; m.p.: 310.3 °C; IR (cm^−1^): 3454, 3319 (NH_2_); ^1^H NMR (DMSO-*d*_6,_ 200 MHz) δ: 8.47 (d, *J* = 2.0 Hz, 1H), 8.28 (dd, *J* = 4.6, 1.5 Hz, 1H), 7.83 (s, 1H), 7.55 (dd, *J* = 7.9, 1.6 Hz, 1H), 7.48 (d, *J* = 8.6 Hz, 1H), 7.32–7.17 (m, 2H), 7.04 (s, 2H), 6.99 (dd, *J* = 7.9, 4.6 Hz, 1H), 3.90 (s, 3H), 3.55 (s, 3H); ^13^C{^1^H} NMR (DMSO-*d_6_*, 50 MHz) δ: 161.3, 152.6, 148.0, 143.3, 143.0, 135.9, 135.8, 130.2, 128.8, 128.1, 126.4, 122.9, 122.3, 118.6, 116.2, 112.5, 110.4, 109.6, 33.5, 31.5; LC-MS: *m/z* [M + H]^+^ calcd for C_20_H_17_ClN_7_^+^ 390.12; found, 390.41.

5-(5-Bromo-1-methyl-1*H*-pyrrolo[2,3-*b*]pyridin-3-yl)-6-(5-chloro-1-methyl-1*H*-indol-3-yl)-1,2,4-triazin-3-amine **(4f)** Yield: 40%; yellow solid; m.p.: 262.1 °C; IR (cm^−1^): 3481, 3285 (NH_2_); ^1^H NMR (DMSO-*d_6,_* 200 MHz) δ: 8.84 (d, *J* = 2.3 Hz, 1H), 8.40 (d, *J* = 2.3 Hz, 1H), 7.68 (s, 1H), 7.58 (d, *J* = 8.7 Hz, 1H), 7.43 (s, 1H), 7.32 (d, *J* = 2.1 Hz, 1H), 7.19 (dd, *J* = 8.7, 2.1 Hz, 1H), 7.14 (s, 2H), 3.86 (s, 3H), 3.61 (s, 3H); ^13^C{^1^H} NMR (DMSO-*d_6_*, 50 MHz) δ: 161.3, 152.0, 146.6, 144.2, 142.8, 135.8, 135.7, 133.5, 131.8, 127.5, 124.8, 122.0, 121.0, 119.6, 113.4, 112.4, 111.0, 108.5, 33.4, 31.9; LC-MS: *m/z* [M + H]^+^ calcd for C_20_H_16_BrClN_7_^+^ 470.02; found, 470.30.

6-(5-Bromo-1-methyl-1*H*-pyrrolo[2,3-*b*]pyridin-3-yl)-5-(5-chloro-1-methyl-1*H*-indol-3-yl)-1,2,4-triazin-3-amine **(5f)** Yield: 39%; yellow solid; m.p.: 334.6 °C; IR (cm^−1^): 3445, 3420 (NH_2_); ^1^H NMR (DMSO-*d_6,_* 200 MHz) δ: 8.39 (d, *J* = 2.1 Hz, 1H), 8.35 (d, *J* = 2.2 Hz, 1H), 7.86 (s, 1H), 7.84 (d, *J* = 2.2 Hz, 1H), 7.55–7.45 (m, 1H), 7.38 (s, 1H), 7.25 (dd, *J* = 8.7, 2.1 Hz, 1H), 7.09 (s, 2H), 3.86 (s, 3H), 3.62 (s, 3H); ^13^C{^1^H} NMR (DMSO-*d_6_*, 50 MHz) δ: 161.3, 152.6, 146.5, 143.3, 142.6, 136.0, 135.7, 131.9, 130.8, 128.1, 126.4, 122.9, 122.2, 120.4, 112.5, 111.6, 110.0, 109.6, 33.6, 31.7; LC-MS: *m/z* [M + H]^+^ calcd for C_20_H_16_BrClN_7_^+^ 470.02; found, 470.30.

6-(5-Fluoro-1-methyl-1*H*-indol-3-yl)-5-(1-methyl-1*H*-pyrrolo[2,3-*b*]pyridin-3-yl)-1,2,4-triazin-3-amine **(4g)** Yield: 41%; yellow solid; m.p.: 267.4 °C; IR (cm^−1^): 3402, 3308 (NH_2_); ^1^H NMR (DMSO-*d_6,_* 200 MHz) δ: 8.74 (dd, *J* = 8.0, 1.6 Hz, 1H), 8.33 (dd, *J* = 4.7, 1.6 Hz, 1H), 7.69 (s, 1H), 7.55 (dd, *J* = 8.9, 4.5 Hz, 1H), 7.34 (s, 1H), 7.20 (dd, *J* = 8.0, 4.7 Hz, 1H), 7.08–6.94 (m, 4H), 3.86 (s, 3H), 3.60 (s, 3H); ^13^C{^1^H} NMR (DMSO-*d_6_*, 50 MHz) δ: 161.3, 156.6 (d, *J* = 262.7 Hz), 152.4, 148.1, 144.1, 143.1, 134.3, 134.0, 132.0, 131.8, 126.6 (d, *J* = 10.8 Hz), 119.5, 117.6, 112.0 (d, *J*= 9.4 Hz), 111.6 (d, *J*= 4.8 Hz), 110.2 (d, *J* = 26.6 Hz), 108.9, 105.1 (d, *J* = 23.5 Hz), 33.4, 31.7; LC-MS: *m/z* [M + H]^+^ calcd for C_20_H_17_FN_7_^+^ 374.15; found, 374.39.

5-(5-Fluoro-1-methyl-1*H*-indol-3-yl)-6-(1-methyl-1*H*-pyrrolo[2,3-*b*]pyridin-3-yl)-1,2,4-triazin-3-amine **(5g)** Yield: 40%; yellow solid; m.p.: 281.4 °C; IR (cm^−1^): 3487, 3410 (NH_2_); ^1^H NMR (DMSO-*d_6,_* 200 MHz) δ: 8.32 (d, *J* = 2.6 Hz, 1H), 8.29 (dd, *J* = 4.6, 1.6 Hz, 1H), 7.84 (s, 1H), 7.57 (dd, *J* = 7.9, 1.6 Hz, 1H), 7.47 (dd, *J* = 9.0, 4.6 Hz, 1H), 7.21 (s, 1H), 7.10 (td, *J* = 9.1, 9.0, 2.6 Hz, 2H), 7.03 (s, 2H), 7.00 (dd, *J* = 7.9, 4.6 Hz, 1H), 3.91 (s, 3H), 3.55 (s, 3H); ^13^C{^1^H} NMR (DMSO-*d_6_*, 50 MHz) δ: 161.3, 160.3, 155.1 (d, *J*= 237.3 Hz), 152.8, 148.0, 143.3, 142.8, 136.0, 134.1, 128.8, 127.7 (d, *J* = 10.8 Hz), 118.6, 116.2, 112.1 (d, *J* = 10.0 Hz), 111.2 (d, *J* = 4.8 Hz), 110.7 (d, *J* = 27.0 Hz), 108.6, 108.5 (d, *J*= 23.5 Hz), 33.7, 31.5; LC-MS: *m/z* [M + H]^+^ calcd for C_20_H_17_FN_7_^+^ 374.15; found, 374.38.

5-(5-Bromo-1-methyl-1*H*-pyrrolo[2,3-*b*]pyridin-3-yl)-6-(5-fluoro-1-methyl-1*H*-indol-3-yl)-1,2,4-triazin-3-amine **(4h)** Yield: 43%; yellow solid; m.p.: 155.6 °C; IR (cm^−1^): 3481, 3288 (NH_2_); ^1^H NMR (DMSO-*d_6,_* 200 MHz) δ: 8.86 (d, *J* = 2.2 Hz, 1H), 8.40 (d, *J* = 2.2 Hz, 1H), 7.71 (s, 1H), 7.56 (dd, *J* = 9.1, 4.5 Hz, 1H), 7.38 (s, 1H), 7.13 (s, 2H), 7.03 (td, *J* = 9.2, 9.1, 2.6 Hz, 1H), 6.96 (dd, *J* = 10.0, 2.6 Hz, 1H), 3.87 (s, 3H), 3.59 (s, 3H); ^13^C{^1^H} NMR (DMSO-*d_6_*, 50 MHz) δ: 161.3, 156.6 (d, *J* = 255.8 Hz), 151.9, 146.6, 144.2, 142.9, 135.7, 134.0, 133.5, 132.0, 129.9 (d, *J* = 12.8 Hz), 121.06, 113.4, 112.0 (d, *J* = 10.2 Hz), 111.4 (d, *J* = 4.5 Hz), 110.3 (d, *J* = 25.7 Hz), 108.5, 105.0 (d, *J* = 23.5 Hz), 33.4, 31.9; LC-MS: *m/z* [M + H]^+^ calcd for C_20_H_16_BrFN_7_^+^ 452.06; found, 452.45.

6-(5-Bromo-1-methyl-1*H*-pyrrolo[2,3-*b*]pyridin-3-yl)-5-(5-fluoro-1-methyl-1*H*-indol-3-yl)-1,2,4-triazin-3-amine **(5h)** Yield: 42%; yellow solid; m.p.: 311.1 °C; IR (cm^−1^): 3479, 3450 (NH_2_); ^1^H NMR (DMSO-*d_6,_* 200 MHz) δ: 8.36 (d, *J* = 2.2 Hz, 1H), 8.23 (dd, *J* = 10.6, 2.6 Hz, 1H), 7.88–7.87 (m, 2H), 7.49 (dd, *J* = 8.9, 4.6 Hz, 1H), 7.35 (s, 1H), 7.11 (td, *J* = 9.1, 2.6 Hz, 1H), 7.06 (s, 2H), 3.87 (s, 3H), 3.62 (s, 3H); ^13^C{^1^H} NMR (DMSO-*d_6_*, 50 MHz) δ: 161.3, 156.8 (d, *J* = 267.1 Hz), 152.8, 146.5, 143.3, 142.4, 135.9, 134.2, 131.9, 130.8, 127.7 (d, *J* = 11.2 Hz), 120.5, 112.1 (d, *J* = 10.2 Hz), 111.6, 111.1 (d, *J* = 26.4 Hz), 110.0, 109.7 (d, *J*= 4.5 Hz), 108.3 (d, *J* = 24.8 Hz), 33.7, 31.7; LC-MS: *m/z* [M + H]^+^ calcd for C_20_H_16_BrFN_7_^+^ 452.06; found, 452.45.

6-(5-methoxy-1-methyl-1*H*-indol-3-yl)-5-(1-methyl-1*H*-pyrrolo[2,3-*b*]pyridin-3-yl)-1,2,4-triazin-3-amine **(4i)** Yield: 47%; yellow solid; m.p.: 217.0 °C; IR (cm^−1^): 3466, 3285 (NH_2_); ^1^H NMR (DMSO-*d_6,_* 200 MHz) δ: 8.72 (dd, *J* = 8.0, 1.6 Hz, 1H), 8.32 (dd, *J* = 4.7, 1.6 Hz, 1H), 7.58 (s, 1H), 7.49–7.39 (m, 1H), 7.30 (s, 1H), 7.19 (dd, *J* = 7.9, 4.7 Hz, 1H), 6.97 (s, 2H), 6.81 (dd, *J* = 8.8, 2.4 Hz, 1H), 6.61 (d, *J* = 2.4 Hz, 1H), 3.83 (s, 3H), 3.58 (s, 3H), 3.44 (s, 3H); ^13^C{^1^H} NMR (DMSO-*d_6_*, 50 MHz) δ: 161.3, 154.2, 152.4, 150.9, 148.1, 144.0, 134.4, 132.4, 131.6, 130.5, 126.7, 119.5, 117.6, 112.2, 111.5, 111.2, 109.0, 102.0, 55.5, 33.3, 31.7; LC-MS: *m/z* [M + H]^+^ calcd for C_21_H_20_N_7_O^+^ 386.17; found, 386.44.

5-(5-Methoxy-1-methyl-1*H*-indol-3-yl)-6-(1-methyl-1*H*-pyrrolo[2,3-*b*]pyridin-3-yl)-1,2,4-triazin-3-amine **(5i)** Yield: 37%; yellow solid; m.p.: 288.2 °C; IR (cm^−1^): 3421, 3329 (NH_2_); ^1^H NMR (DMSO-*d_6,_* 200 MHz) δ: 8.28 (dd, *J* = 4.6, 1.5 Hz, 1H), 7.77 (d, *J* = 2.4 Hz, 2H), 7.62 (dd, *J* = 7.9, 1.5 Hz, 1H), 7.33 (d, *J* = 8.9 Hz, 1H), 7.21 (s, 1H), 7.00 (dd, *J* = 7.9, 4.6 Hz, 1H), 6.94 (s, 2H), 6.84 (dd, *J* = 8.8, 2.5 Hz, 1H), 3.86 (s, 3H), 3.68 (s, 3H), 3.54 (s, 3H); ^13^C{^1^H} NMR (DMSO-*d_6_*, 50 MHz) δ: 161.2, 155.3, 153.2, 148.0, 143.3, 143.0, 134.8, 132.5, 130.1, 128.9, 127.6, 118.7, 116.2, 112.7, 111.5, 110.7, 109.7, 105.1, 55.8, 33.5, 31.4; LC-MS: *m/z* [M + H]^+^ calcd for C_21_H_20_N_7_O^+^ 386.17; found, 386.43.

5-(5-Bromo-1-methyl-1*H*-pyrrolo[2,3-*b*]pyridin-3-yl)-6-(5-methoxy-1-methyl-1*H*-indol-3-yl)-1,2,4-triazin-3-amine **(4j)** Yield: 48%; yellow solid; m.p.: 197.6 °C; IR (cm^−1^): 3481, 3447 (NH_2_); ^1^H NMR (DMSO-*d_6,_* 200 MHz) δ: 8.82 (d, *J* = 2.2 Hz, 1H), 8.39 (d, *J* = 2.2 Hz, 1H), 7.60 (s, 1H), 7.42 (d, *J* = 8.8 Hz, 1H), 7.35 (s, 1H), 7.07 (s, 2H), 6.80 (dd, *J* = 8.9, 2.4 Hz, 1H), 6.59 (d, *J* = 2.4 Hz, 1H), 3.84 (s, 3H), 3.57 (s, 3H), 3.45 (s, 3H); ^13^C{^1^H} NMR (DMSO-*d_6_*, 50 MHz) δ: 161.3, 154.2, 151.8, 146.5, 144.1, 143.5, 135.8, 133.3, 132.4, 130.5, 126.6, 121.0, 113.3, 112.3, 111.5, 111.0, 108.7, 101.9, 55.6, 33.3, 31.9; LC-MS: *m/z* [M + H]^+^ calcd for C_21_H_18_BrN_7_O^+^ 465.32; found, 464.40.

6-(5-Bromo-1-methyl-1*H*-pyrrolo[2,3-*b*]pyridin-3-yl)-5-(5-methoxy-1-methyl-1*H*-indol-3-yl)-1,2,4-triazin-3-amine **(5j)** Yield: 48%; yellow solid; m.p.: 269.8 °C; IR (cm^−1^): 3481, 3291 (NH_2_); ^1^H NMR (DMSO-*d_6,_* 200 MHz) δ: 3.61 (s, 3H), 3.68 (s, 3H), 3.83 (s, 3H), 6.84 (dd, *J* = 8.9, 2.5 Hz, 1H), 6.97 (s, 2H), 7.36–7.33 (m, 2H), 7.68 (d, *J* = 2.5 Hz, 1H), 7.81 (s, 1H), 7.87 (d, *J* = 2.2 Hz, 1H), 8.35 (d, *J* = 2.2 Hz, 1H); ^13^C{^1^H} NMR (DMSO-*d_6_*, 50 MHz) δ: 31.7, 33.5, 55.8, 105.0, 109.7, 110.3, 111.5, 111.6, 112.8, 120.5, 127.5, 130.8, 131.8, 132.6, 134.7, 142.7, 143.2, 146.4, 153.1, 155.3, 161.2; LC-MS: *m/z* [M + H]^+^ calcd for C_21_H_18_BrN_7_O^+^ 465.32; found, 466.34.

### 3.2. Software Overview

All the molecular modeling operations were executed within the Molecular Operating Environment (MOE) suite (version 2019.01) [56] [. The proteins were downloaded as PDB files from the Protein Data Bank (PDB) [57], and their preparation for the computational studies was executed by exploiting the dedicated tools available in the MOE package, as well as the generation of the homology models used for the molecular docking studies. The ligands were properly prepared for computational handling with the tools of the QUACPAC package available in the OpenEye suite [58]. The molecular docking calculations were executed with the program PLANTS [59], setting PLANTSCHEMPLP as the scoring function. The hardware exploited for all the computational studies was a 20-CPU Linux Workstation (Intel i9-9820X).

### 3.3. PDK1-4 Kinase Assay

The in vitro inhibitory activity of tested compounds on recombinant human PDK1-4 isoforms (Abcam, Cambridge, MA, USA) was evaluated by using the ADPGlo™ Kinase Assay kit from Promega (Promega Corporation, Madison, WI, USA) following the instructions of the manufacturer, as previously described [44].

### 3.4. Heat Shock Protein 90 (HSP90) Inhibition Assay 

The in vitro inhibitory activity of tested compounds on recombinant human HSP90 was assessed by The Transcreener™ ADP kit (Bellbrook Labs, Madison, WI, USA) according to the manufacturer’s protocol as previously described [44]. 

### 3.5. Experiments with Cultured Human Cancer Cells 

All tested compounds were dissolved in DMSO just before the experiment, and a calculated amount of drug solution was added to the cell growth medium to a final solvent concentration of 0.5%, which had no detectable effects on cell viability. Dichloroacetate (DCA), 2,2-dichloroacetophenone (DAP) and gemcitabine hydrochloride were purchased by Merck KGaA (Darmstadt, Germany).

### 3.6. Cell Cultures

Human pancreatic PSN-1 and BxPC-3 carcinoma cell lines were obtained by American Type Culture Collection (ATCC, Rockville, MD, USA). Cell lines were maintained in the logarithmic phase at 37 °C in a 5% carbon dioxide atmosphere using RPMI-1640 medium (EuroClone) containing 10% fetal calf serum (EuroClone, Milan, Italy), antibiotics (50 units/mL penicillin and 50 μg/mL streptomycin) and 2 mM l-glutamine.

### 3.7. MTT Assay

The growth inhibitory effect on tumor cells was evaluated by means of MTT assay. Briefly, 5 × 10^3^ cells/well were seeded in 96-well microplates in a growth medium (100 μL). After 24 h, the medium was removed and replaced with a fresh one containing the compound to be studied at the appropriate concentration. Triplicate cultures were established for each treatment. After 72 h, each well was treated with 10 μL of a 5 mg/mL MTT saline solution, and following 5 h of incubation, 100 μL of a sodium dodecyl sulfate (SDS) solution in HCl 0.01 M was added. After overnight incubation, cell growth inhibition was detected by measuring the absorbance of each well at 570 nm using a Bio-Rad 680 microplate reader. The mean absorbance for each drug dose was expressed as a percentage of the control untreated well absorbance and plotted vs. drug concentration. IC_50_ values, the drug concentrations that reduce the mean absorbance at 570 nm to 50% of those in the untreated control wells, were calculated by the four-parameter logistic (4-PL) model. The evaluation was based on means from at least four independent experiments.

### 3.8. Spheroid Cultures

Spheroid cultures were obtained by seeding 2.5 × 10^3^ PSN-1 cancer cells/well in a round-bottom non-treated tissue culture 96-well plate (Greiner Bio-one, Kremsmünster, Austria) in phenol red-free RPMI-1640 medium (Sigma Chemical Co, St. Louis, MO, USA) containing 10% fetal calf serum and supplemented with 20% methylcellulose stock solution. 

### 3.9. Acid Phosphatase (APH) Assay

An APH-modified assay was used for determining cell viability in 3D spheroids, as previously described [60]. Briefly, the pre-seeded spheroids were treated with fresh medium containing the compound to be studied at the appropriate concentration (range 2.5–200 μM). Triplicate cultures were established for each treatment. After 72 h, each well was treated with 100 μL of the assay buffer (0.1 M sodium acetate, 0.1% Triton-X-100, supplemented with ImmunoPure p-nitrophenyl phosphate; Sigma Chemical Co.) and, following 3 h of incubation, 10 μL of 1 M NaOH solution were added. The inhibition of the cell growth induced by the tested compounds was detected by measuring the absorbance of each well at 405 nm, using a Bio-Rad 680 microplate reader. The mean absorbance for each drug dose was expressed as a percentage of the control untreated well absorbance (T/C) and plotted vs. drug concentration. IC_50_ values, the drug concentrations that reduce the mean absorbance at 405 nm 50% of those in the untreated control wells, were calculated by the four-parameter logistic (4-PL) model. The evaluation was based on means from at least four independent experiments.

## 4. Conclusions

To the best of our knowledge, very few studies reported small molecules as PDK inhibitors employed for the treatment of metabolic altered types of cancer, such as PDAC. PDKs are a family of kinases with a key role of control in cellular energy metabolism, whose deregulation led to cancer formation sustaining tumour cell proliferation, invasion, angiogenesis, apoptosis resistance and metabolic switch. Among the existing therapies, chemotherapy regimens represent the standard treatment for most patients; unfortunately, PDAC tumor metabolism alteration has been proven to be associated with chemoresistance. Therefore, there is an urgent need to develop novel therapeutic strategies to treat PDAC. In the present study, we presented the synthesis and pharmacological evaluation of a new series of 1,2,4-triazine derivatives **4a-j**, **5a-j**, with promising PDKs enzymatic inhibition, especially with preferential activity towards the PDK1 and PDK4 isoforms. In particular, compounds **4d**, **4f**, **4h**, **5d**, **5f**, **5h**, and **5j** totally hampered the enzymatic activity of PDK1 at the tested concentration of 1.5 µM, at the same time compounds **4d**, **5d**, **5f**, **5h** and **5j** were also able to suppress the enzymatic activity of PDK4. On the other hand, all the tested derivatives were scarcely effective against PDK2 and PDK3. The IC_50_ values on the PDK1 isoforms were also calculated for all derivatives, confirming their efficiency in decreasing the PDK1 catalytic activity, with IC_50_ values ranging from 0.04 to 0.33 µM. In addition, considering the high degree of similarity shared by PDK1 and HSP90, the most promising compounds were also screened for their ability to block the enzymatic activity on HSP90. The results showed that the selected derivatives were able to completely suppress the activity of HSP0, except for compound **5j**. Considering these results, molecular docking analyses were performed on both, PDK1 and HSP90. All the new synthesized compounds were docked into the orthosteric ATP- binding site. In the case of PDK1, binding mode of these new compounds was experimentally validated, using the ligand-based homology modeling technique, which showed that all derivatives interact through hydrogen bond with Asp318 by nitrogen triazine central core, whereas the bicyclic portions are placed in the inner part of the binding site interacting with solvent exposed area. Moreover, the methyl groups of the indole/azaindole interact with hydrophobic area. A very similar binding mode was also observed for HSP0; therefore, the key differences in the activities could be explained by the dynamic processes, which allows some molecules to better fit in the pocket than others. In vitro antiproliferative activities against KRAS-wild-type and mutant PDAC cells BXPC-3 and PSN-1 were assessed by using an MTT assay. Interestingly, all derivatives were much more effective against KRAS-mutant PSN-1 showing promising cytotoxic activity, with IC_50_ values in the micromolar range. In order to further confirm the antiproliferative activity found, the compounds were tested on 3D spheroids of PDAC which better simulate the pathophysiological tumor specific microenvironment. Interestingly, also in 3D models, the compounds were much more effective in the KRAS-mutant PSN-1, confirming the cytotoxic activity of the new triazine derivatives.

## Figures and Tables

**Table 1:** 3-Amino-1,2,4-triazine derivatives **4a–j, 5a–j** and their relative yields.

Cpd	R	R^1^	Overall Yield%	4 (%) 5 (%)	Cpd	R	R^1^	Overall Yield%	4 (%) 5 (%)
**4a**	H	H	80	42	**4f**	Br	Cl	79	40
**5a**				38	**5f**				39
**4b**	Br	H	86	44	**4g**	H	F	81	41
**5b**				42	**5g**				40
**4c**	H	Br	94	49	**4h**	Br	F	85	43
**5c**				45	**5h**				42
**4d**	Br	Br	92	48	**4i**	H	OCH_3_	84	47
**5d**				44	**5i**				37
**4e**	H	Cl	87	46	**4j**	Br	OCH_3_	96	48
**5e**				41	**5j**				48

**Table 2:** Inhibition of isolated PDK1 isoform or HSP90. IC_50_ values were calculated by a four-parameter analyzed logistic model (*p* < 0.05). DCA and DAP were reported as reference compounds. Data represent mean values from 3 independent experiments. S.D. = standard deviation.

Cpd	IC_50_ (µM) ± S.D. PDK1	IC_50_ (µM) ± S.D. HSP90	Cpd	IC_50_ (µM) ± S.D. PDK1	IC_50_ (µM) ± S.D. HSP90
**4a**	0.30 ± 0.10	-	**5a**	0.32 ± 0.10	-
**4b**	0.33 ± 0.05	-	**5b**	0.21 ± 0.07	-
**4c**	0.26 ± 0.07	-	**5c**	0.27 ± 0.08	-
**4d**	0.04 ± 0.02	0.06 ± 0.02	**5d**	0.05 ± 0.02	0.07 ± 0.01
**4e**	0.29 ± 0.06	-	**5e**	0.32 ± 0.10	-
**4f**	0.12 ± 0.04	0.10 ± 0.03	**5f**	0.08 ± 0.02	0.11 ± 0.04
**4g**	0.19 ± 0.04	-	**5g**	0.29 ± 0.10	-
**4h**	0.06 ± 0.02	-	**5h**	0.05 ± 0.03	0.09 ± 0.03
**4i**	0.33 ± 0.10	-	**5i**	0.31 ± 0.07	-
**4j**	0.18 ± 0.06	-	**5j**	0.04 ± 0.02	0.18 ± 0.04
**DCA**	15,890 ± 256	29,740 ± 558	**DAP**	212 ± 48	698 ± 83

**Table 3:** Antiproliferative activity of tested compounds against PSN-1 and BxPC3 cancer cells evaluated in 2D and 3D cytotoxicity assay.

Cpd	IC_50_ (µM) ± S.D. BxPC-3 ^a^	IC_50_ (µM) ± S.D. PSN-1 ^a^	IC_50_ (µM) ± S.D. BxPC-3 ^b^	IC_50_ (µM) ± S.D. PSN-1 ^b^
**4a**	15.7 ± 2.2	11.3 ± 2.0	141.3 ± 0.1	97.5 ± 2.8
**4b**	23.5 ± 3.2	19.5 ± 2.9	223.7 ± 14.8	188.5 ± 13.8
**4c**	9.5 ± 1.7	7.8 ± 1.5	76.8 ± 5.1	55.8 ± 3.5
**4d**	30.2 ± 6.2	23.4 ± 3.7	175.5 ± 15.7	102.8 ± 9.1
**4e**	32.3 ± 8.2	26.5 ± 4.3	159.5 ± 11.4	59.6 ± 4.5
**4f**	18.4 ± 1.4	11.3 ± 1.8	168.9 ± 12.4	162.2 ± 6.8
**4g**	6.5 ± 1.1	4.5 ± 1.3	71.5 ± 5.2	65.5 ± 6.8
**4h**	22.3 ± 2.4	17.5 ± 2.8	187.5 ± 14.4	77.8 ± 3.8
**4i**	31.2 ± 5.5	26.5 ± 4.0	189.4 ± 10.9	139.5 ± 7.5
**4j**	30.5 ± 3.4	27.8 ± 3.8	159.7 ± 10.5	102.8 ± 8.7
**5a**	27.2 ± 3.7	22.2 ± 2.7	169.5 ± 9.4	78.8 ± 8.5
**5b**	9.4 ± 1.3	5.2 ± 0.9	68.5 ± 2.5	73.6 ± 6.8
**5c**	13.7 ± 2.9	9.2 ± 1.9	156.5 ± 11.7	78.4 ± 5.8
**5d**	26.7 ± 6.1	21.3 ± 3.5	198.5 ± 14.2	215.6 ± 13.6
**5e**	16.5 ± 3.8	14.2 ± 3.1	199.7 ± 16.3	197.5 ± 12.4
**5f**	22.4 ± 3.2	21.3 ± 4.4	229.4 ± 17.2	212.8 ± 14.4
**5g**	19.8 ± 3.2	15.2 ± 4.6	79.7 ± 11.4	129.5 ± 11.8
**5h**	0.05 ± 0.03	27.4 ± 5.1	208.3 ± 12.7	132.5 ± 5.9
**5i**	0.31 ± 0.07	14.9 ± 3.2	153.4 ± 10.3	112.5 ± 9.1
**5j**	0.04 ± 0.02	0.1 ± 0.03	79.5 ± 5.4	89.7 ± 5.7
**DCA**	>1000	>1000	36200 ± 125	29800 ± 357
**DAP**	16.5 ± 0.9	10.20 ± 1.6	78.2 ± 6.2	87.4 ± 8.9
**Gemcitabine**	0.020 ± 0.003	0.10 ± 0.04	159.5 ± 5.6	102.6 ± 6.3

^a^ Cells (5 × 10^3^·well) were treated for 72 h with increasing concentrations of tested compounds. Cytotoxicity was assessed by MTT test. ^b^ Spheroids (2.5 × 10^3^ cells/well) were treated for 72 h with increasing concentrations of tested compounds. The growth-inhibitory effect was evaluated by means of the acid phosphatase (APH) test. IC_50_ values were calculated by a four-parameter logistic model (*p* < 0.05). Gemcitabine, DCA and DAP were reported as reference compounds. Data represent mean values from at least 3 independent experiments. S.D. = standard deviation.

## Data Availability

Not applicable.

## Supplementary Materials

The following supporting information can be downloaded at: https://www.mdpi.com/article/10.3390/md21050288/s1, Figures S1–S20: Chromatogram and MS spectrum of compound (**4a**–**j** and **5a**–**j**).
