# Supplementary material for: Structural Manipulations of Marine Natural Products Inspire a New Library of 3-Amino-1,2,4-Triazine PDK Inhibitors Endowed with Antitumor Activity in Pancreatic Ductal Adenocarcinoma
Source: Mar Drugs. 2023 May 4;21(5):288. doi: 10.3390/md21050288 (PMC10224441; doi:10.3390/md21050288)
Supplement: Supplementary file 1 [file marinedrugs-21-00288-s001.zip › marinedrugs-2324893-supplementary.pdf]

Article

Structural manipulations of marine natural products inspire a new library of 3-amino-1,2,4-triazine PDK inhibitors endowed with anti-tumor activity in pancreatic ductal adenocarcinoma

Daniela Carbone[1], Michele De Franco[2], Camilla Pecoraro[1], Davide Bassani[3], Matteo Pavan[3], Stella Cascioferro[1], Barbara Parrino[1], Girolamo Cirrincione[1], Stefano Dall'Acqua[2], Stefania Sut[2], Stefano Moro[3], Valentina Gandin[2*] and Patrizia Diana[1*]

1 Department of Biological, Chemical, and Pharmaceutical Sciences and Technologies (STEBICEF), University of Palermo, Via Archirafi 32, 90123, Palermo, Italy; daniela.carbone@unipa.it; camilla.pecoraro@unipa.it; stellamaria.cascioferro@unipa.it; barbara.parrino@unipa.it; girolamo.cirrincione@unipa.it; patrizia.diana@unipa.it
2 Department of Pharmaceutical and Pharmacological Sciences, University of Padova, Via Marzolo 5, 35128, Padova, Italy; michele.defranco@studenti.unipd.it; stefano.dallacqua@unipd.it; stefania.sut@studenti.unipd.it; valentina.gandin@unipd.it
3 Molecular Modeling Section (MMS), Department of Pharmaceutical and Pharmacological Sciences, University of Padova, 35131 Padova, Italy; davide.bassani.1@phd.unipd.it; matteo.pavan.7@phd.unipd.it; stefano.moro@unipd.it
* Correspondence: patrizia.diana@unipa.it; valentina.gandin@unipd.it

Content	Figures
MS spectra	S1-S20

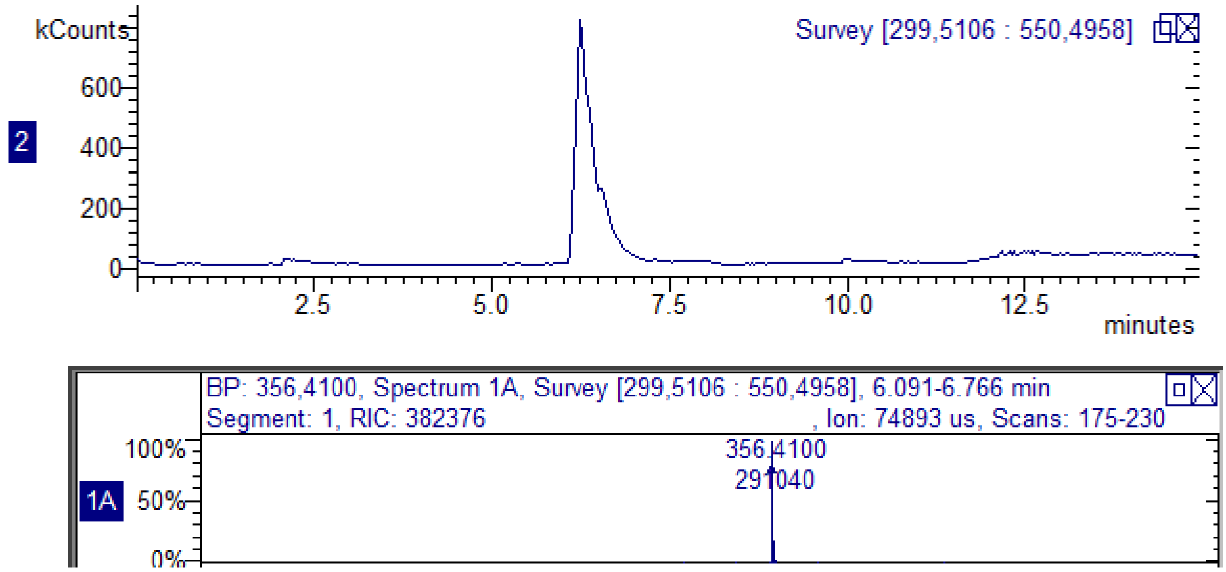

Figure S1. Chromatogram and MS spectrum of compound **4a.**

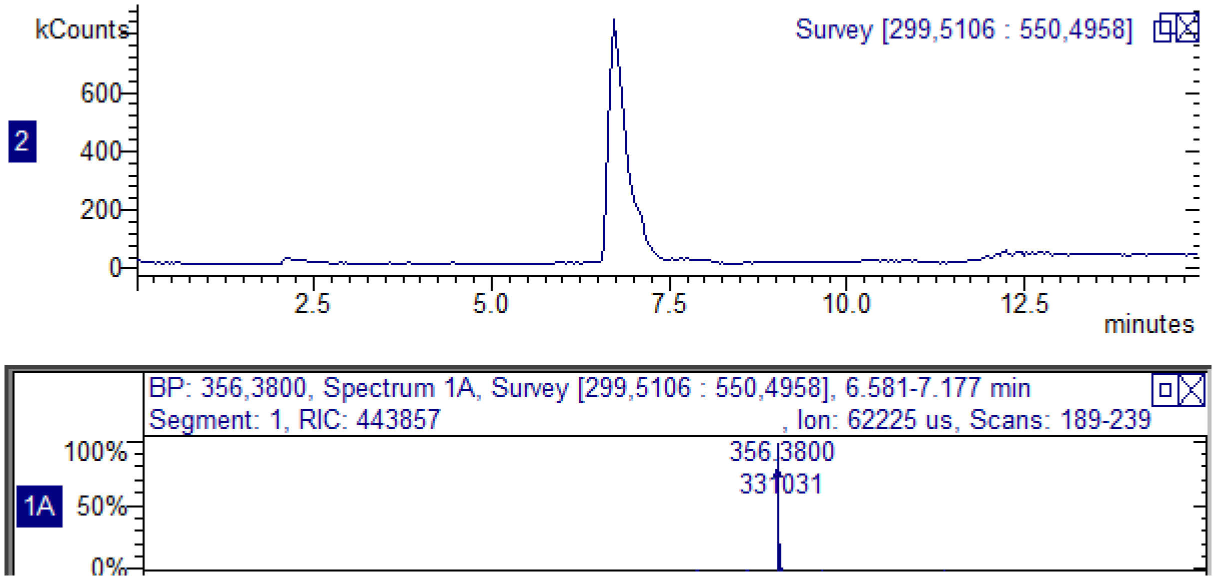

Figure S2. Chromatogram and MS spectrum of compound **5a.**

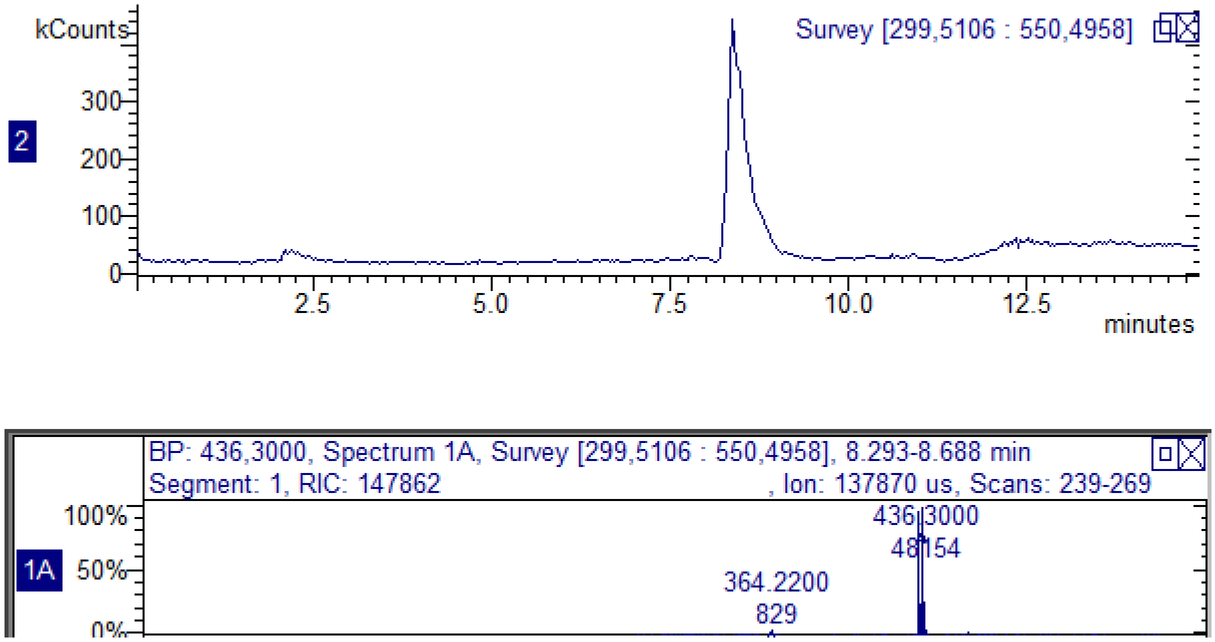

Figure S3. Chromatogram and MS spectrum of compound **4b.**

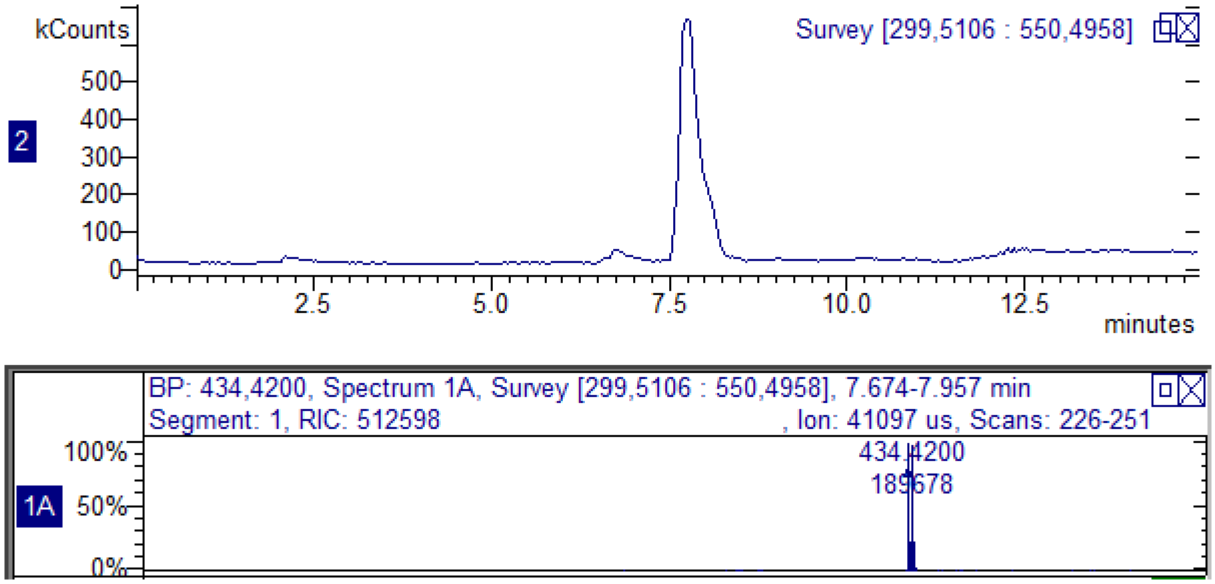

Figure S4. Chromatogram and MS spectrum of compound **5b.**

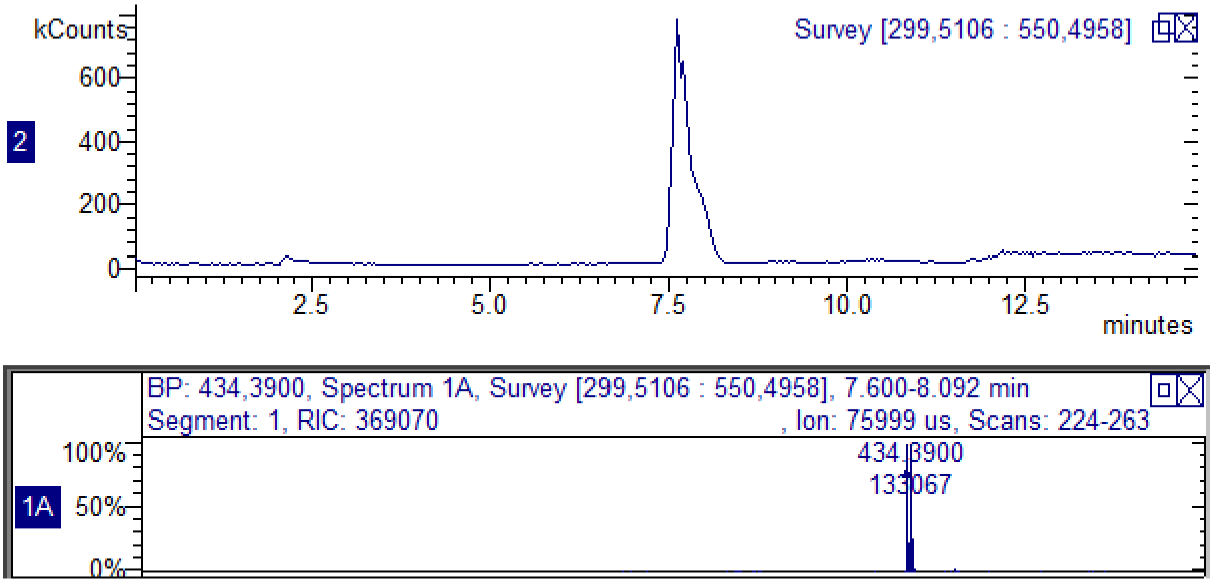

Figure S5. Chromatogram and MS spectrum of compound **4c.**

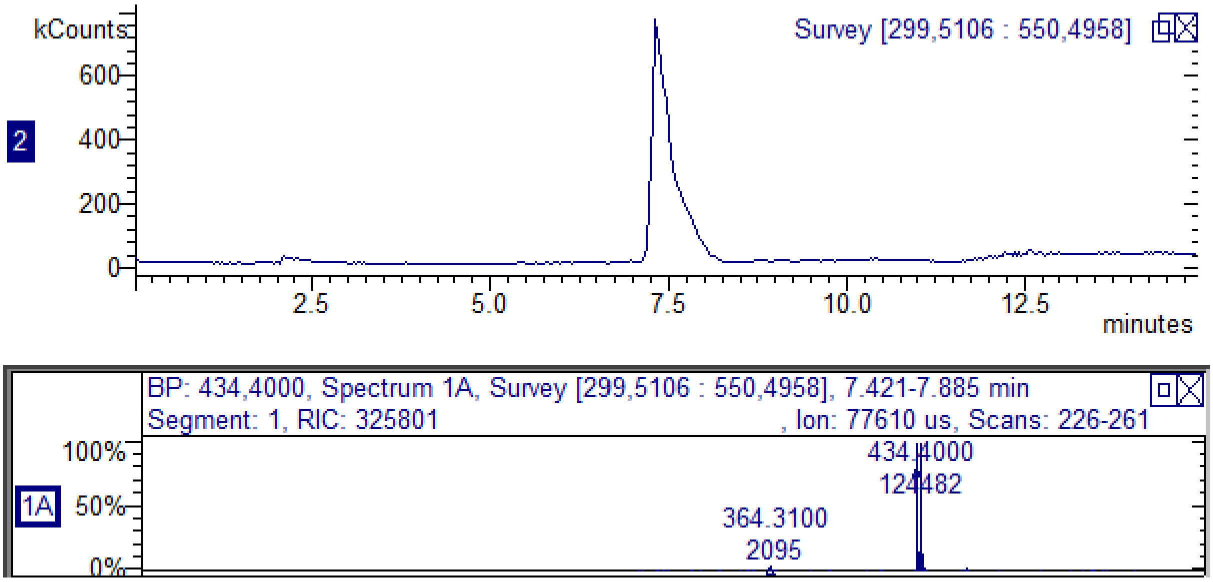

Figure S6. Chromatogram and MS spectrum of compound **5c.**

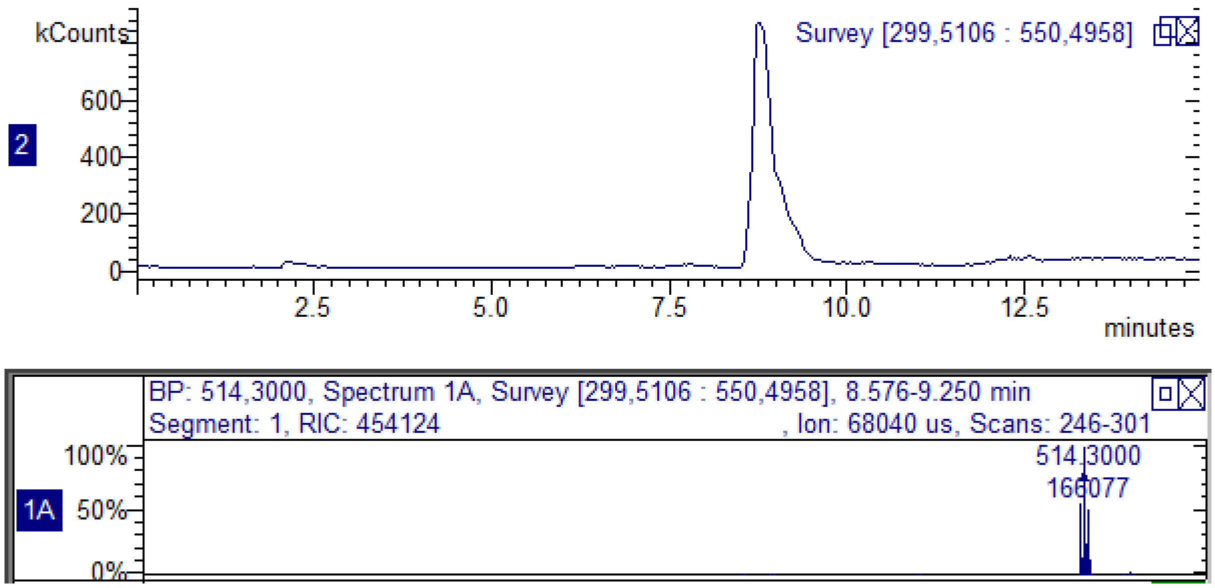

Figure S7. Chromatogram and MS spectrum of compound **4d.**

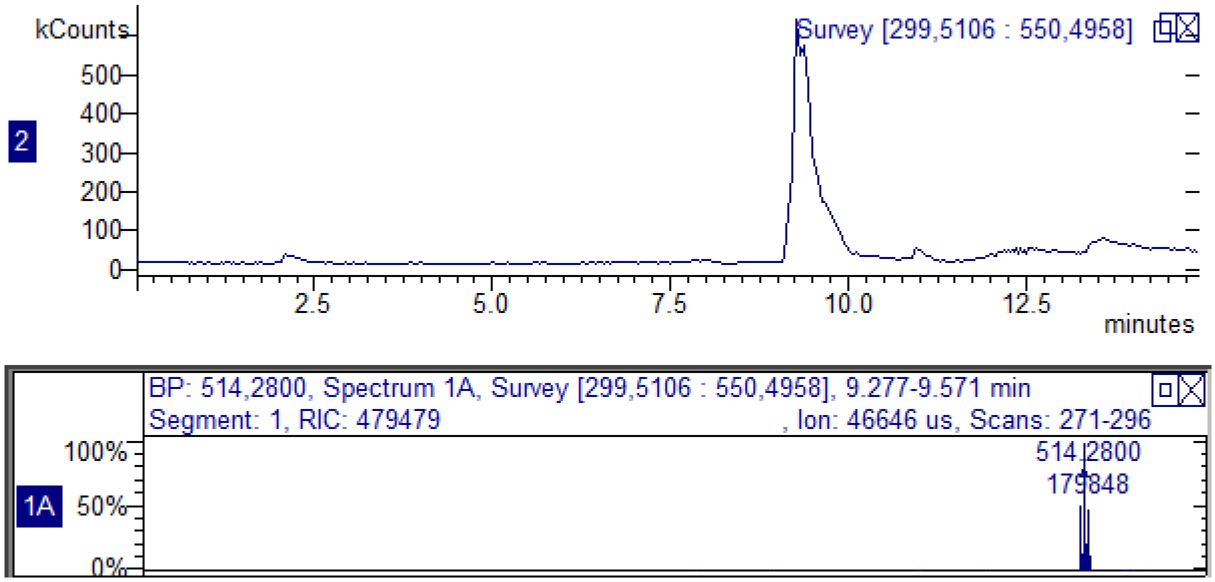

Figure S8. Chromatogram and MS spectrum of compound **5d.**

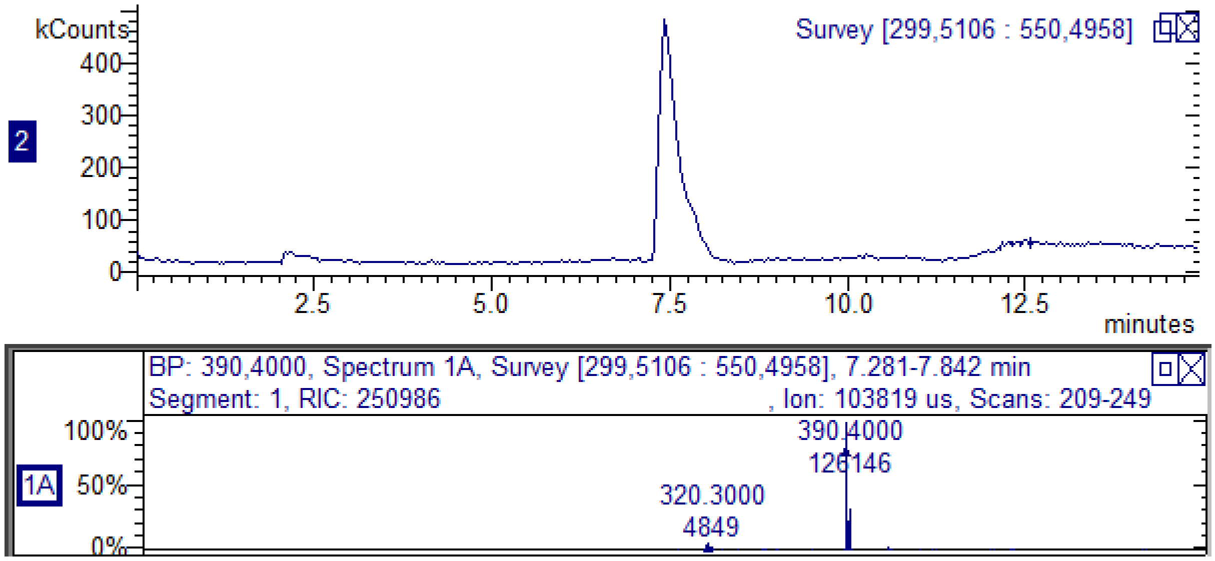

Figure S9. Chromatogram and MS spectrum of compound **4e.**

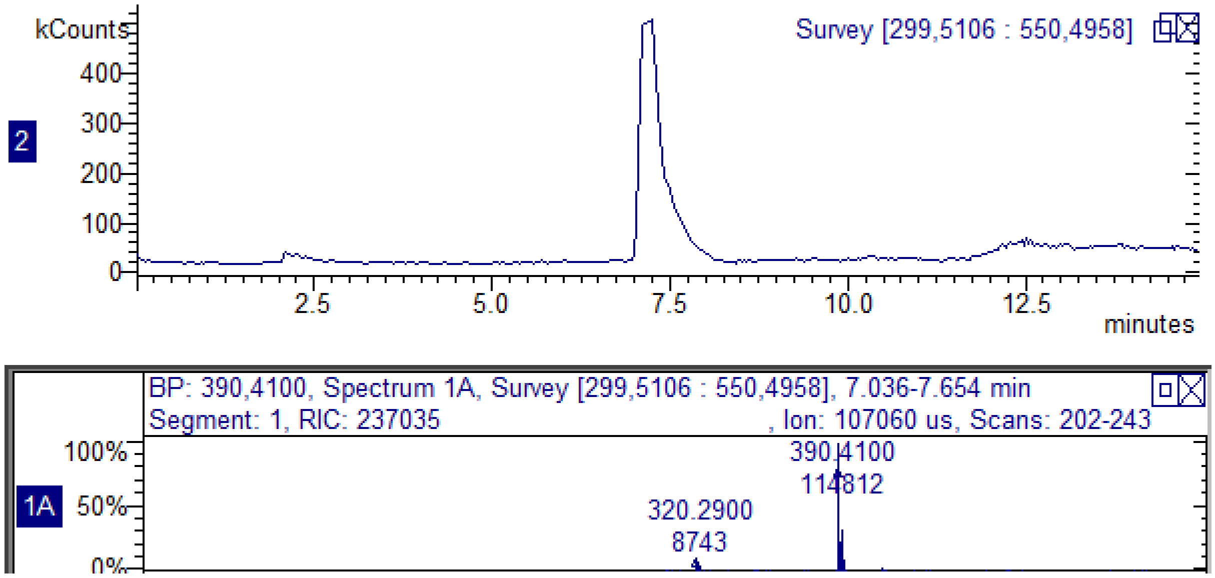

Figure S10. Chromatogram and MS spectrum of compound **5e.**

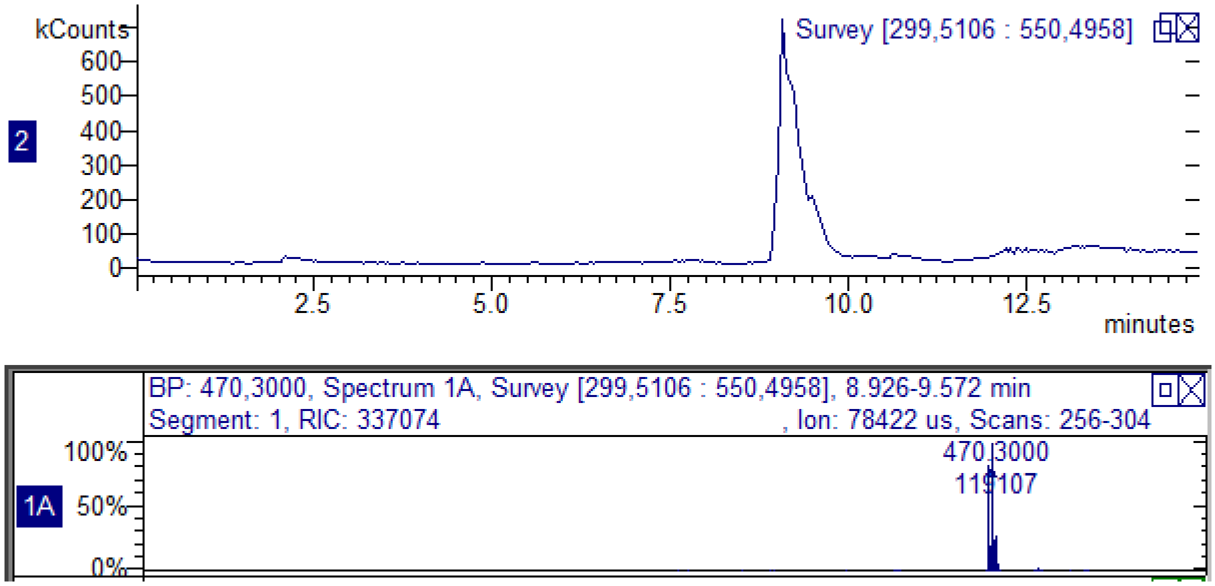

Figure S11. Chromatogram and MS spectrum of compound **4f.**

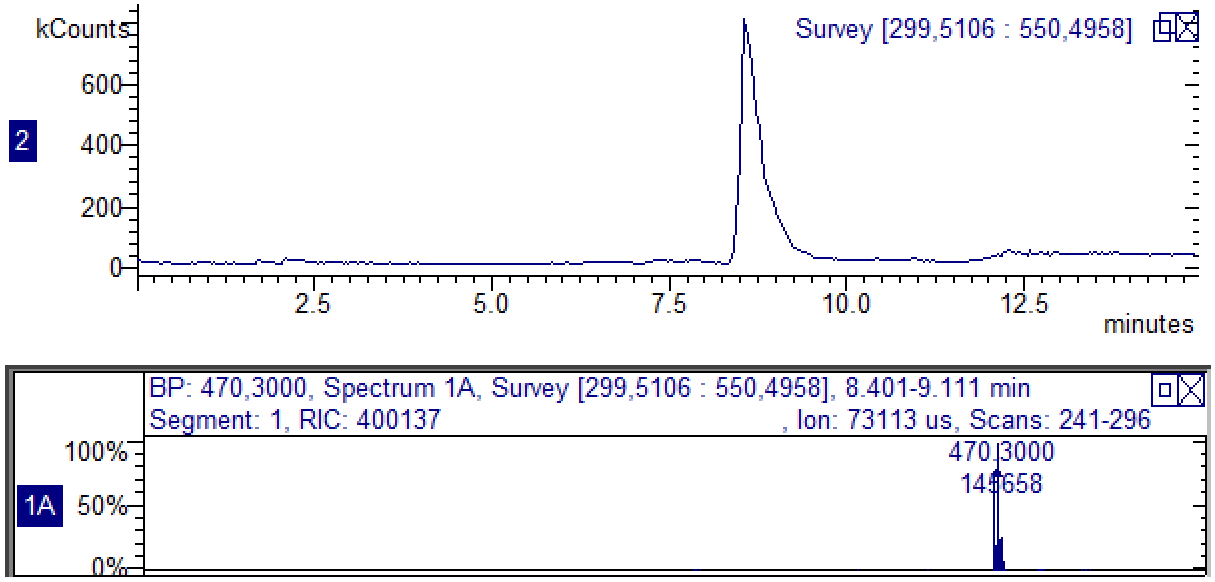

Figure S12. Chromatogram and MS spectrum of compound **5f.**

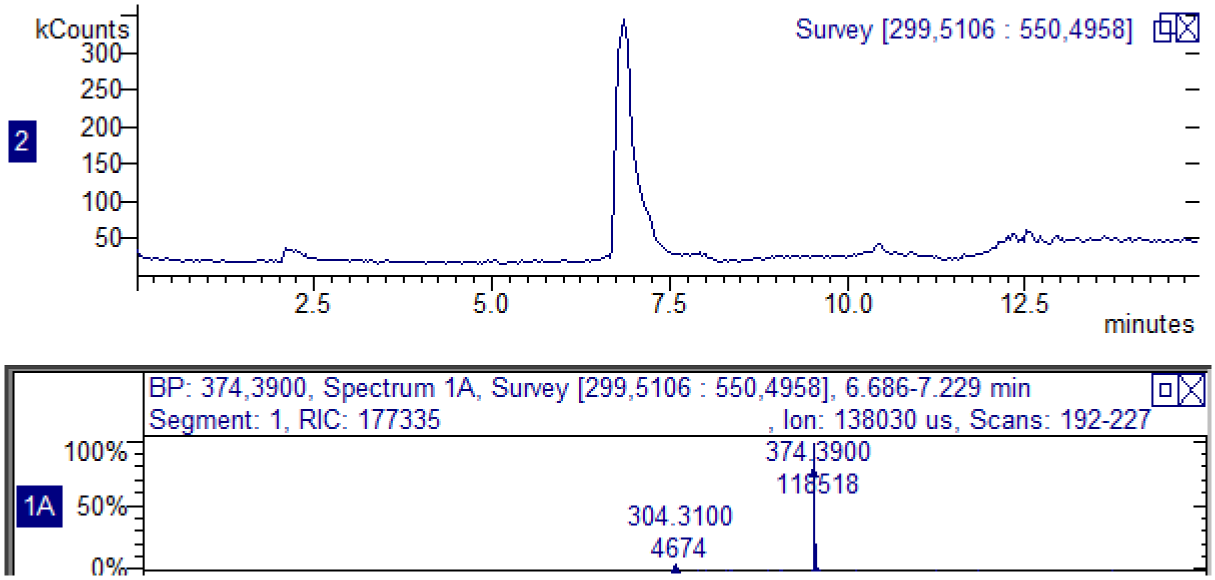

Figure S13. Chromatogram and MS spectrum of compound **4g.**

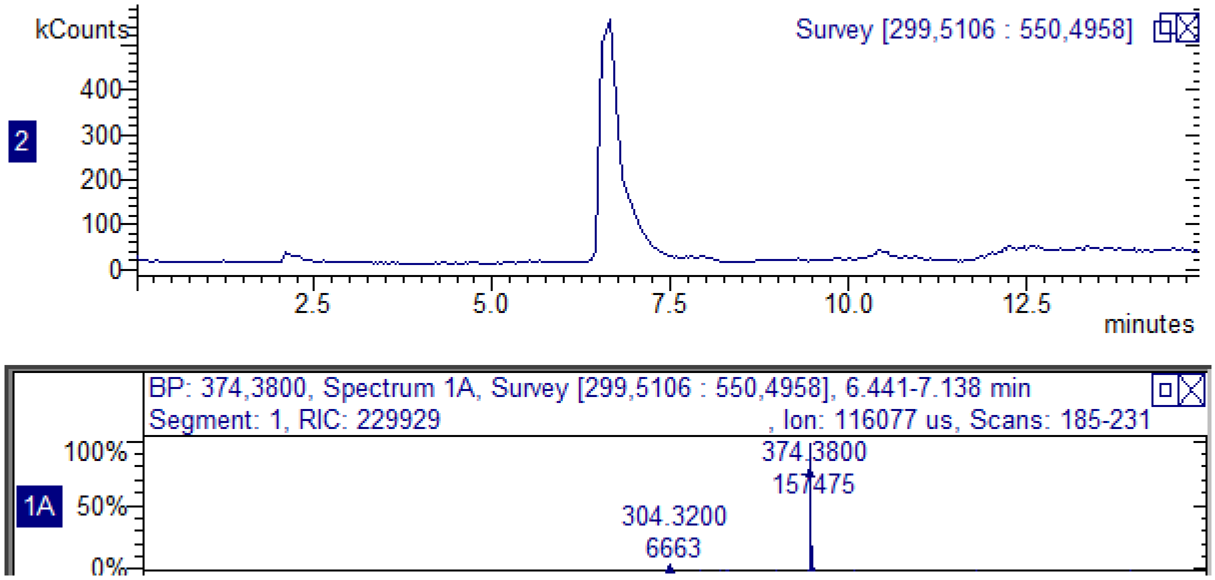

Figure S14. Chromatogram and MS spectrum of compound **5g.**

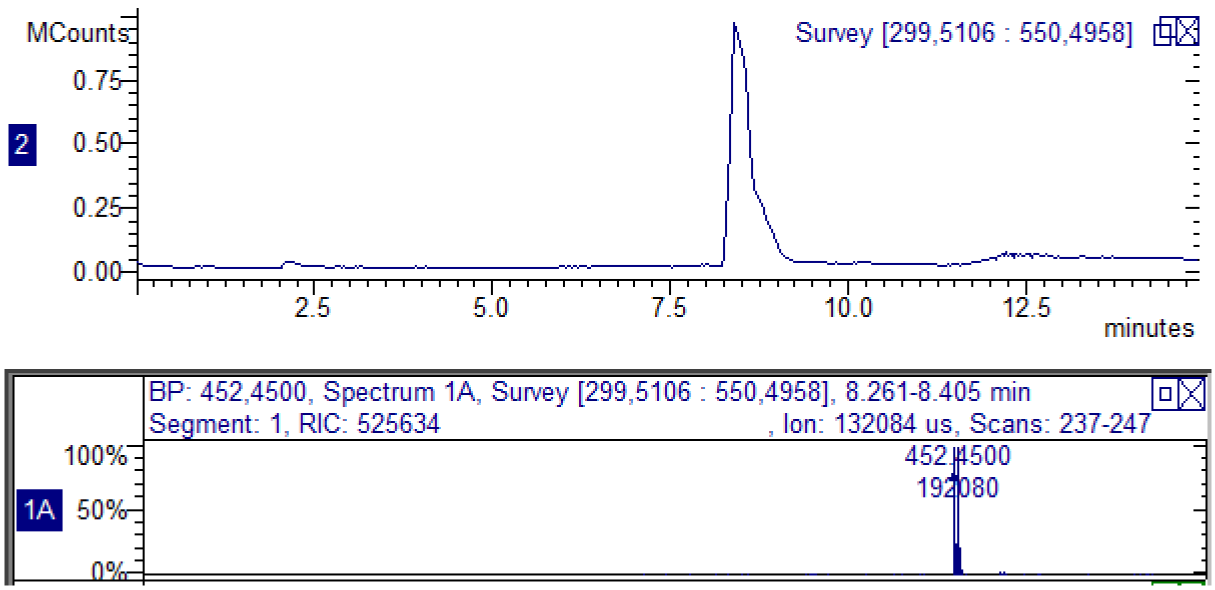

Figure S15. Chromatogram and MS spectrum of compound **4h.**

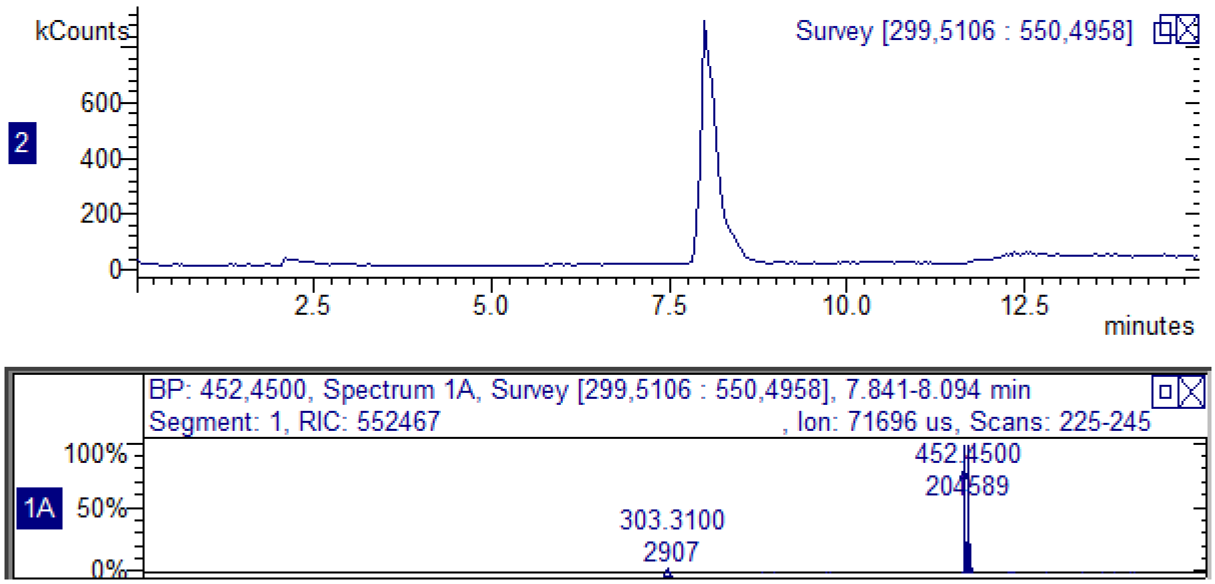

Figure S16. Chromatogram and MS spectrum of compound **5h.**

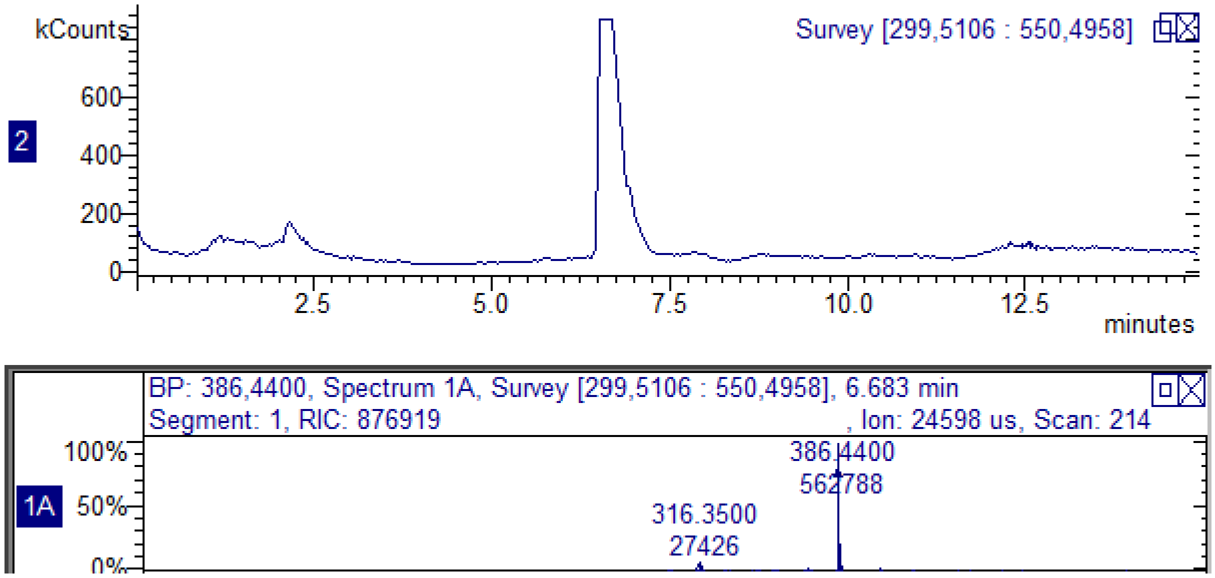

Figure S17. Chromatogram and MS spectrum of compound **4i.**

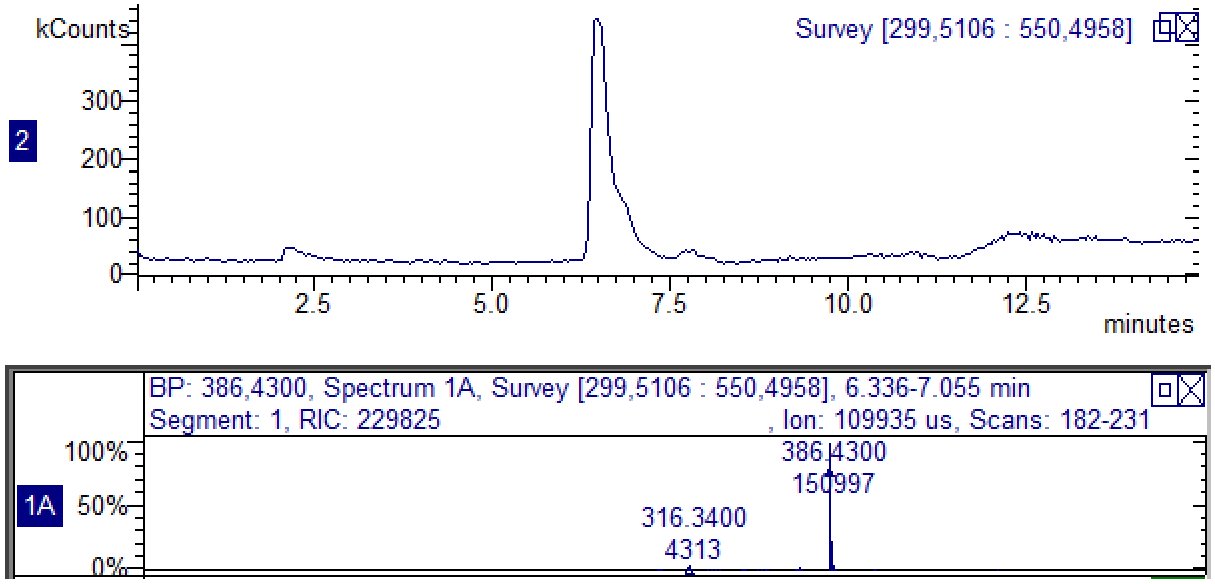

Figure S18. Chromatogram and MS spectrum of compound **5i.**

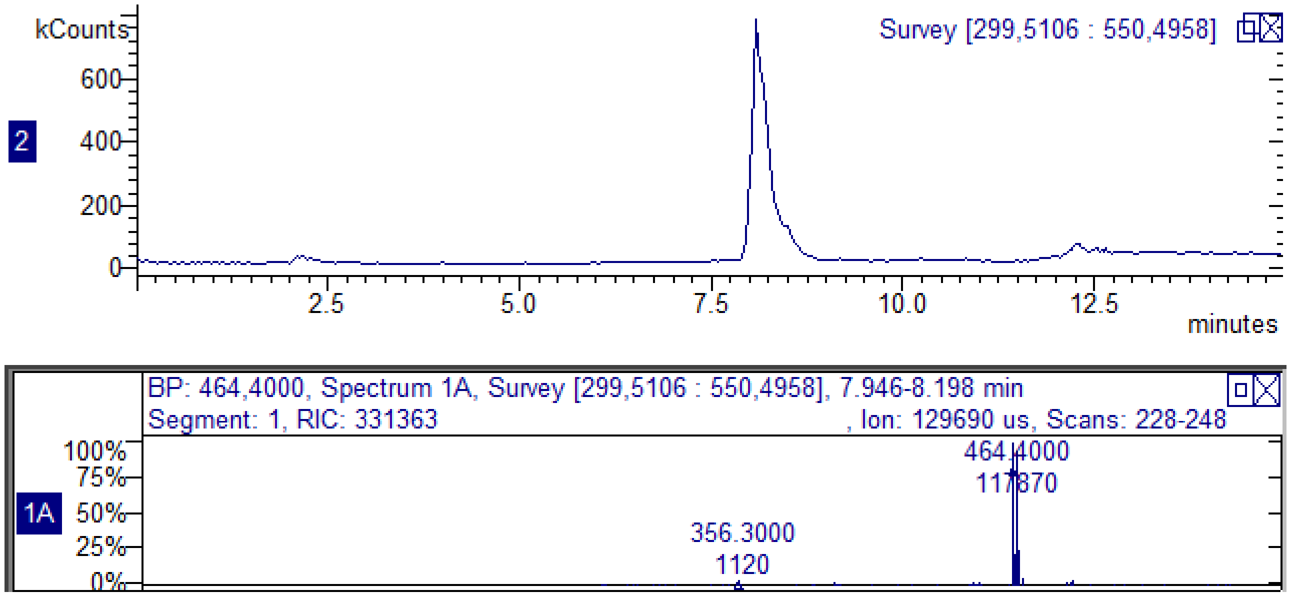

Figure S19. Chromatogram and MS spectrum of compound **4j.**

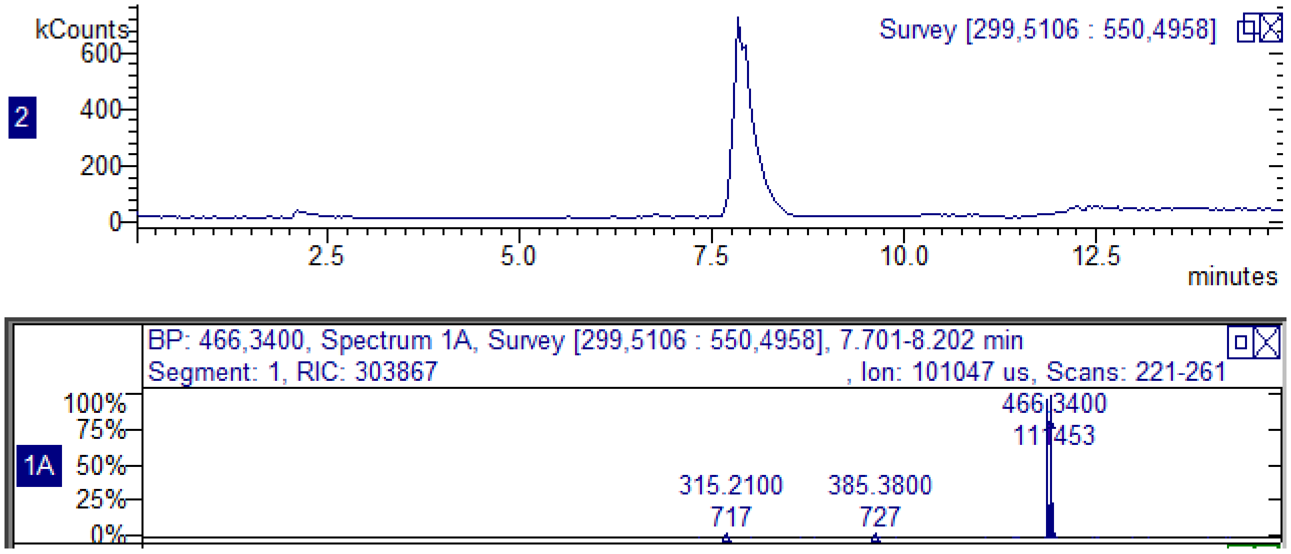

Figure S20. Chromatogram and MS spectrum of compound **5j.**
